# Comprehensive Deep Mutational Scanning Reveals the Immune-Escaping Hotspots of SARS-CoV-2 Receptor-Binding Domain Targeting Neutralizing Antibodies

**DOI:** 10.3389/fmicb.2021.698365

**Published:** 2021-07-15

**Authors:** Keng-Chang Tsai, Yu-Ching Lee, Tien-Sheng Tseng

**Affiliations:** ^1^National Research Institute of Chinese Medicine, Ministry of Health and Welfare, Taipei, Taiwan; ^2^Ph.D. Program in Medical Biotechnology, College of Medical Science and Technology, Taipei Medical University, Taipei, Taiwan; ^3^TMU Research Center of Cancer Translational Medicine, Taipei Medical University, Taipei, Taiwan; ^4^Ph.D. Program for Cancer Molecular Biology and Drug Discovery, College of Medical Science and Technology, Taipei Medical University, Taipei, Taiwan; ^5^Ph.D. Program in Biotechnology Research and Development, College of Pharmacy, Taipei Medical University, Taipei, Taiwan; ^6^TMU Biomedical Commercialization Center, Taipei Medical University, Taipei, Taiwan; ^7^Institute of Molecular Biology, National Chung Hsing University, Taichung, Taiwan

**Keywords:** SARS-CoV-2, COVID-19, binding stability, hotspots, neutralization, antibody, immunity

## Abstract

The rapid spread of SARS-CoV-2 has caused the COVID-19 pandemic, resulting in the collapse of medical care systems and economic depression worldwide. To combat COVID-19, neutralizing antibodies have been investigated and developed. However, the evolutions (mutations) of the receptor-binding domain (RBD) of SARS-CoV-2 enable escape from neutralization by these antibodies, further impairing recognition by the human immune system. Thus, it is critical to investigate and predict the putative mutations of RBD that escape neutralizing immune responses. Here, we employed computational analyses to comprehensively investigate the mutational effects of RBD on binding to neutralizing antibodies and angiotensin-converting enzyme 2 (ACE2) and demonstrated that the RBD residues K417, L452, L455, F456, E484, G485, F486, F490, Q493, and S494 were consistent with clinically emerging variants or experimental observations of attenuated neutralizations. We also revealed common hotspots, Y449, L455, and Y489, that exerted comparable destabilizing effects on binding to both ACE2 and neutralizing antibodies. Our results provide valuable information on the putative effects of RBD variants on interactions with neutralizing antibodies. These findings provide insights into possible evolutionary hotspots that can escape recognition by these antibodies. In addition, our study results will benefit the development and design of vaccines and antibodies to combat the newly emerging variants of SARS-CoV-2.

## Introduction

SARS-CoV-2, which causes viral pneumonia in humans, is the cause of COVID-19 (Lai et al., 2020). Under an electron microscope, the virus exhibits crown-like morphology (“corona”) and is thus named coronavirus (Gui et al., 2017). The World Health Organization declared COVID-19 as a pandemic. In April 2021, there were 142.5 million confirmed cases of COVID-19, including 3,043,707 deaths (daily online worldwide data about COVID-19^1^). Common symptoms of SARS-CoV-2 infection include diarrhea, dry cough, fever, nasal congestion, respiratory problems, and sore throat (Baj et al., 2020). In severe cases, kidney failure, severe acute respiratory syndrome, and pneumonia may ensue, eventually leading to death (Lai et al., 2020).

SARS-CoV-2, a single-stranded positive-sense enveloped RNA virus, consists of an RNA sequence of approximately 30,000 bases (Naqvi et al., 2020). This viral genome has 10 open reading frames (ORF) (Tsai et al., 2020). Of these, ORF1ab encodes polyprotein lab (pp1ab), which is cleaved by the proteases 3CL^*pro*^ and PL^*pro*^ to yield multiple proteins associated with viral RNA replication and transcription (Graham et al., 2008; Moustaqil et al., 2021) as well as 16 non-structural proteins, creating the replication–transcription complex of SARS-CoV-2 (Romano et al., 2020). In addition, ORFs 2–10 encode four structural proteins: spike (S), membrane (M), nucleocapsid (N), and envelope (E). The N protein is critical for packing the RNA genome, and the S, M, and E proteins are essential for viral coating. The S protein is a large oligomeric transmembrane protein responsible for the entry of the virus into the host cell (Lan et al., 2020). It comprises two functional domains: S1 and S2; the S1 domain comes into contact directly with the angiotensin-converting enzyme 2 (ACE2) receptor on the host cell (Wrapp et al., 2020), whereas the S2 domain mediates cell membrane fusion (Walls et al., 2020; Wrapp et al., 2020).

SARS-CoV-2 enters the host cell through ACE2; thus, the S protein partly determines its transmissibility and infectivity (Hoffmann et al., 2020). The receptor-binding domain (RBD) of the S1 subunit directly interacts with ACE2 (Lan et al., 2020; Yang et al., 2020). Thus, some antiviral drugs targeting RBD were developed. Small molecules, such as chloroquine, hydroxychloroquine, ivermectin, and azithromycin, have been reported to target the S protein–ACE2 interface (Pandey et al., 2020; Batalha et al., 2021; Mirtaleb et al., 2021). Moreover, novel drug-like compounds DRI-C23041 (Rajgor et al., 2020) and DRI-C91005 (Lan et al., 2020) have been observed to inhibit the S protein–ACE2 interaction, with low micromolar activity. The S protein is immunogenic; hence, several approaches have targeted it for viral neutralization. Neutralizing antibodies targeting RBD have also been developed (Pinto et al., 2020; Rogers et al., 2020; Xiaojie et al., 2020; Liu et al., 2021; Lu et al., 2021). Some antibody-based antiviral therapeutics have demonstrated high specificity, potency, and modularity. However, RNA viruses continually change through mutations, leading to the emergence of new variants (Pachetti et al., 2020; Nagy et al., 2021; Wang et al., 2021). This antigenic evolution leading to RBD mutations overcomes the established neutralizing antibody immunity (Eguia et al., 2021; Greaney et al., 2021a). It is therefore critical to systematically monitor the antigenic evolution and investigate viral mutations that can impair the immune response conferred by neutralizing antibodies.

Here, we comprehensively estimated the RBD mutations that destabilize the binding of five representative neutralizing antibodies: the H11-D4 and VH1-2-15 nanobodies, MR17 and SR4 sybodies, and P2B-2F6 Fab, which target RBD’s receptor-binding motif. We employed complex structures retrieved from the Protein Data Bank to calculate binding stability through detailed mutational scanning, in which a single residue was replaced by all other 20 amino acids in RBD to systematically investigate the hotspots that affect binding. The resulting heatmaps demonstrated that mutations at R403, K417, G447, N448, Y449, N450, L452, Y453, L455, F456, E484, G485, F486, Y489, F490, P491, L492, Q493, S494, Y495, and G496 were unfavorable for binding with antibodies. Notably, the E484K and L452R mutants are also present in the P.1 viral lineage. Moreover, F456 variants have reduced binding to neutralizing antibodies, and L455, F486, and F490 have substantial antigenic effects. The N501Y mutant is present in emerging viral lineages, such as B1.1.7 and B.1.351. All the aforementioned clinical and experimental reports support our findings. Thus, the interactive residues of RBD (Y449, L452, L455, E484, Y489, F490, L492, Q493, and S494) identified in this study can be hotspots for further antibody engineering or vaccine developments to combat potential variants of SARS-CoV-2.

## Materials and Methods

### Preparation of Protein Structures

The structures of the SARS-CoV-2 S protein in complex with nanobodies, sybodies, Fabs, and ACE2 were retrieved from the Protein Data Bank (PDB IDs: 6M0J, 6YZ5, 7BWJ, 7C8V, 7C8W, and 7L5B). The CHARMm Polar H forcefield was applied to all complex structures before computations.

### Calculation of Mutational Binding Stability

The mutational binding stability of RBD with its targets was estimated by Discovery Studio (DS) 3.5 (Accelrys, San Diego, CA, United States), MutaBind2 (Zhang et al., 2020), FoldX (Schymkowitz et al., 2005), and mCSM-PPI2 (Rodrigues et al., 2019). For the prediction in DS, the Calculate Mutation Energy (Binding) protocol was used to estimate the mutational binding stability. The complex structure was used as the “input typed molecule,” and the complexed partners of RBD were employed as the “ligand chain.” Additionally, interactive residues of RBD, making direct contact with its targets (within a maximum distance of 5 Å from the targets’ interface), were selected for a mutational study. Furthermore, a single mutation was used as “mutation sites,” and all 20 amino acids were chosen as the “mutations” parameter.” Additionally, the dielectric constant [The dielectric constant of a molecular interior corresponds to the measure of electric potential energy. Therefore, the induced polarization is stored within a given volume of substance under the action of an electric field. It is expressed as the ratio of the dielectric permittivity of the material to that of a vacuum or dry air (Ilic, 2001)], solvent dielectric constant, maximum structures to save, and maximum number of mutants were set to 10, 80, 25, and 25, respectively. All other parameters were set to default. For the prediction in FoldX, the binding stability was determined according to a previous report (Teng et al., 2021). In addition, the calculations in MutaBind2 and mCSM-PPI2 were followed by the online prediction instructions. The mutational binding stability resulted from the calculations of the effect of mutations on the binding affinity. We performed combinatorial scanning mutagenesis in protein complexes, depending on the selected mutation sites. For each single mutant, the differences in the free energy of binding between the wild-type and mutated structures are calculated. Mutational energy is the total free energy difference between the wild-type and mutated structures. It is calculated as a weighted sum of the VDW, electrostatic, entropy, and non-polar terms. Accordingly, the negative and positive values of mutational energies represent the stabilized and destabilized binding stabilities.

### Generation of Heatmaps of Mutational Binding Energy

The binding energy values upon single mutations were subjected to heatmap generation by using Excel. The residues for mutations were used as the *y*-axis, and the amino acid types of single mutations were used as the *x*-axis. The binding energy values of all mutations were selected for conditional formatting. The blue and red colors are used to indicate the values of binding energies, respectively, with the gradient to a range of cells for the color scales.

### Ligplot Analyses

The molecular interactions of each complex structure of the S protein with its targets were analyzed using a ligplot (Wallace et al., 1995; Laskowski and Swindells, 2011), and its DIMPLOT module was employed for plotting protein–protein and domain–domain interactions.

## Results

### Mutational Binding Stability of RBD Variants Interacting With the H11-D4 Nanobody

**FIGURE 1:**
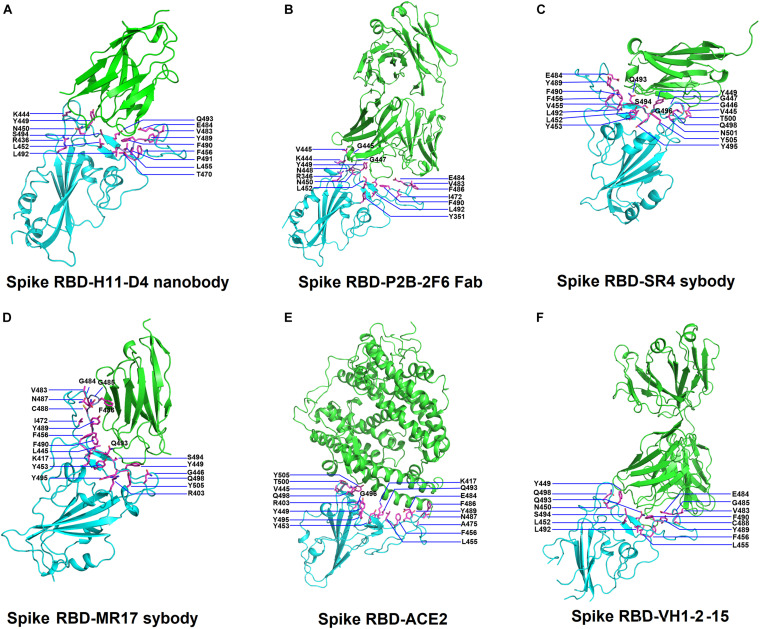
Structures of SARS-Cov-2 receptor-binding domain (RBD) in complex with neutralizing antibodies and angiotensin-converting enzyme 2 (ACE2). **(A)** The complex structure of RBD with H11-D4 nanobody (PDB ID: 6YZ5). **(B)** The complex structure of RBD with P2B-2F6 Fab (PDB ID: 7BWJ). **(C)** The complex structure of RBD with SR4 sybody (PDB ID: 7C8V). **(D)** The complex structure of RBD with MR17 sybody (PDB ID: 7C8W). **(E)** The complex structure of RBD with ACE2 (PDB ID: 6M0J). **(F)** The complex structure of RBD with the VH1-2-15 nanobody (PDB ID: 7L5B). In all panels, the protein structures are shown in ribbons and colored in cyan (RBD) and green (antibodies) with the interactive residues (side chains) presented in sticks (magenta).

**FIGURE 2:**
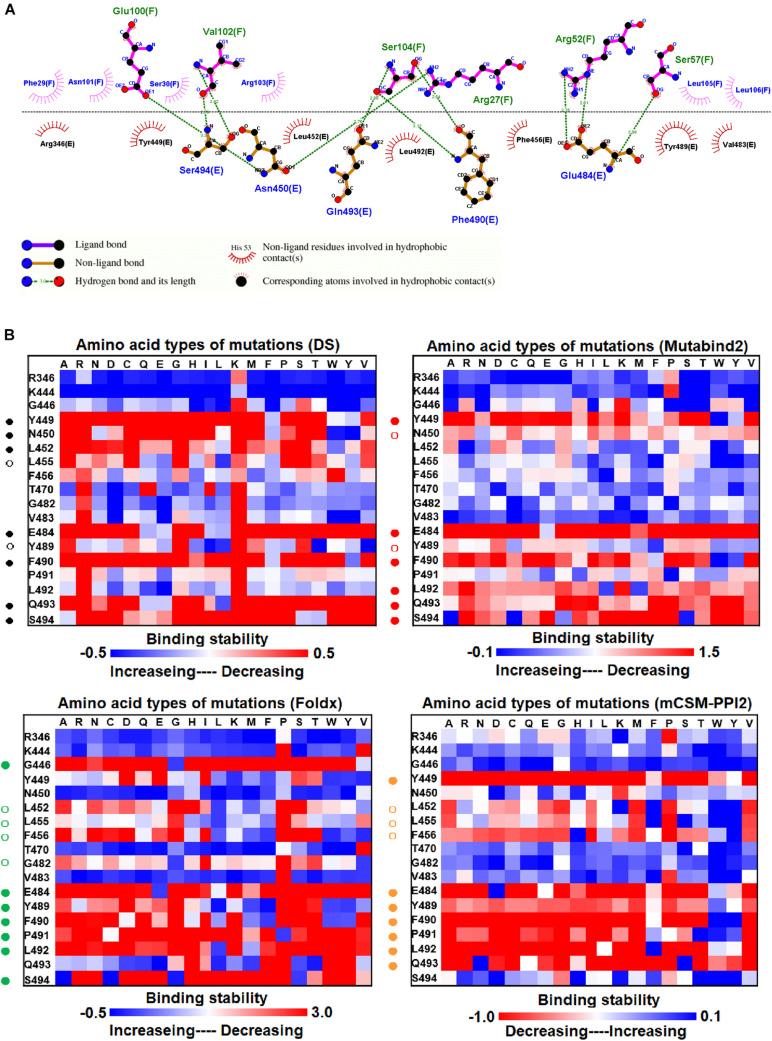
The mutational binding stabilities of RBD variants interacting to H11-D4 nanobody. **(A)** The molecular interactions of SARS-CoV-2 RBD with H11-D4 nanobody analyzed by ligplot. The chains E and F correspond to RBD and H11-D4 nanobody, respectively. **(B)** The heatmap of interactive residues of RBD derived from the calculated mutational binding stabilities by using Discovery Studio 3.5 (DS), Mutabind2, FoldX, and mCSM-PPI2. The boxes of each mutations were colored with the gradient of a range between blue (stabilized binding) and red (destabilized binding). In all panels, the solid and hollow circles denote significant and moderate decreases of the binding stabilities, respectively.

Before investigating the effects of RBD mutations on the binding of antibodies, we searched for the dissolved complex structure of RBD with antibodies in the Protein Data Bank. The H11-D4 nanobody has high affinity to the S protein and blocks its attachment to ACE2. The structure of the H11-D4 nanobody in complex with RBD is illustrated in Figure 1A. The epitope of RBD to H11-D4 was analyzed with a ligplot and is illustrated in Figure 2A. The RBD residues N450, E484, F490, Q493, and S494 predominantly form hydrogen bonds, and residues R346, Y449, L452, F456, V483, Y489, and L492 make hydrophobic contacts with the H11-D4 nanobody. These interactive residues were further mutated into all 20 amino acids to estimate their effects on the binding stability against the H11-D4 nanobody. Additionally, RBD residues that make direct contact with the H11-D4 nanobody (within a maximum distance of 5 Å from the nanobody interface) were selected for the mutational study. The resultant binding stability of the RBD variants was plotted into a heatmap, with the *y*-axis as the residues mutated and the *x*-axis as the amino acid types of single mutations. The calculated binding stability values are colored with the gradient of a range between blue (stabilized binding) and red (destabilized binding). The results revealed that mutations at Y449, N450, L452, E484, Y489, F490, P491, L492, Q493, and S494 were mostly not favorable for binding stability (Figure 2B and Table 1).

**TABLE 1:** Mutation sites destabilizing SARS-CoV-2 RBD binding to neutralizing antibodies.

Destabilizing mutations on SARS-CoV-2 RBD																							
	
	R403	K417	G447	N448	Y449^∗^	N450	L452^∗^	Y453	L455^c*^	F456^c^	E484^c*^	G485^c^	F486^c^	C488	Y489^∗^	F490^c*^	P491	L492^∗^	Q493^∗^	S494^∗^	Y495	G496	
H11-D4 (nanobody)					○	○	○		○		○				○	○	○	○	○	○			
VH1-2-15(nanobody)					○					○	○	○		○	○	○		○		○		○	
SR4 (sybody)			○		○		○	○	○	○					○	○		○	○	○		○	
MR17 (sybody)	○	○						○	○		○		○	○	○	○			○		○		
P2B-2F6 (Fab)			○	○	○	○	○				○					○							

**Identified variants**																							

^a^United Kingdom (Nigeria); B.1.525											**E484K**												
^a^United Kingdom B.1.1.7											**E484K**									**S494P**			**N501Y**
^a^United States (New York); B.1.526											**E484K**												
^a^United States (California) B.1.427, B.1.429							**L452R**																
^a^United States (New York); B.1.526.1							**L452R**																
^a^India B.1.617, B.1.617.1 B.1.617.2, B.1.617.3							**L452R**				**E484Q**												
^a^Brazil, P.1		**K417N**									**E484K**												**N501Y**
^a^Brazil, P.2					_‘_						**E484K**												
^a^South Africa, B.1.351		**K417N**									**E484K**												**N501Y**
^a^Japan, P.1		**K417T**									**E484K**												**N501Y**
^b^Peru, C.37							**L452R**									**F490S**							**N501Y**

*Hotspots.

*^*a*^U.S. Department of Health and Human Services (https://www.cdc.gov/coronavirus/2019-ncov/cases-updates/variant-surveillance/variant-info.html).*

*^*b*^European Centre for Disease Prevention and Control (https://www.ecdc.europa.eu/en/covid-19/variants-concern).*

*^*c*^Mutations at other structurally adjacent sites in the RBD’s receptor-binding ridge can also have substantial antigenic effects.*

*The bold values are residue number in SARS-COV-2 RBD.*

### Mutational Effects on the Binding Stability of RBD With VH1-2-15 Nanobody

**FIGURE 3:**
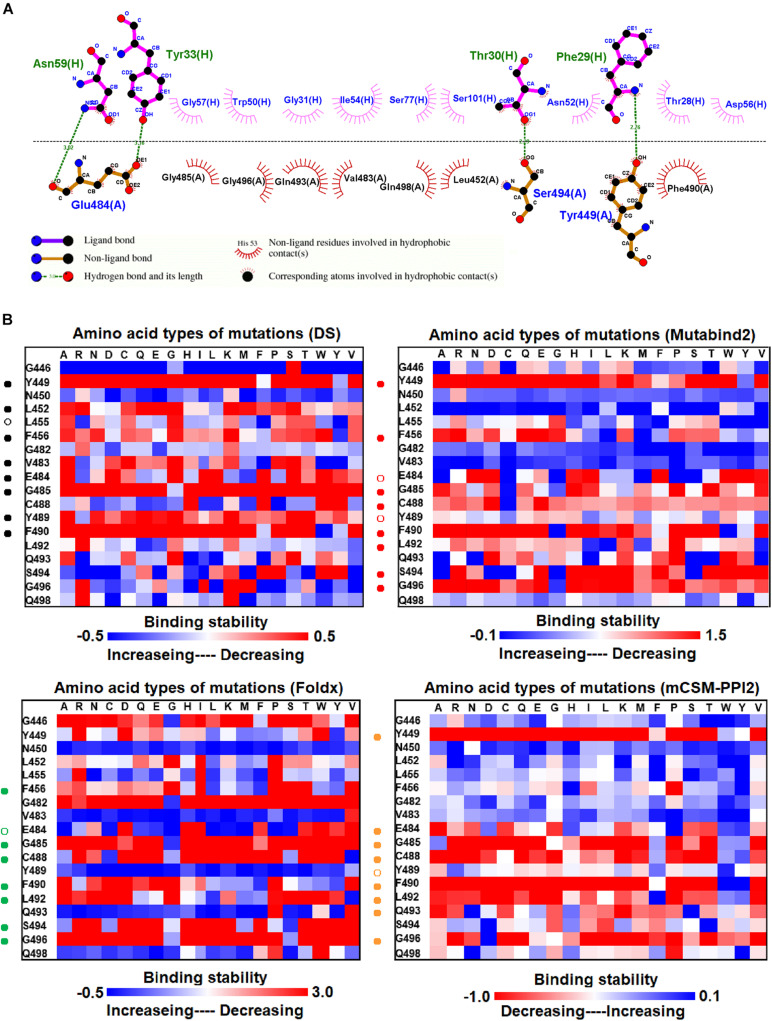
The mutational binding stabilities of RBD variants interacting to VH1-2-15 nanobody. **(A)** The molecular interactions of SARS-CoV-2 RBD with VH1-2-15 nanobody analyzed by ligplot. The chains A and H correspond to RBD and VH1-2-15 nanobody, individually. **(B)** The heatmap of interactive residues of RBD derived from the calculated mutational binding stabilities by using Discovery Studio 3.5 (DS), Mutabind2, FoldX, and mCSM-PPI2. The boxes of each mutations were colored with the gradient of a range between blue (stabilized binding) and red (destabilized binding). In all panels, the solid and hollow circles indicate significant and moderate decreases of the binding stabilities, respectively.

We investigated the mutational effects of RBD on binding stability by using a distinct complex structure. The RBD structure in complex with the VH1-2-15 nanobody is illustrated in Figure 1F. Ligplot analyses illustrate that the RBD residues Y449, E484, and S494 form hydrogen bonds, and L452, V483, G485, Q493, G496, Q498, and F490 make hydrophobic contacts with VH1-2-15 (Figure 3A). These interactive residues of RBD were further subjected to mutational calculations to estimate their binding stability with VH1-2-15. The result indicated that mutations at the residues Y449, F456, E484, G485, C488, F490, L492, S494, and G496 were predominantly unfavorable for binding to the VH1-2-15 nanobody (Figure 3B and Table 1).

### Variations in Mutational Binding Stability of RBD Mutants Targeting SR4 and MR17 Sybodies

**FIGURE 4:**
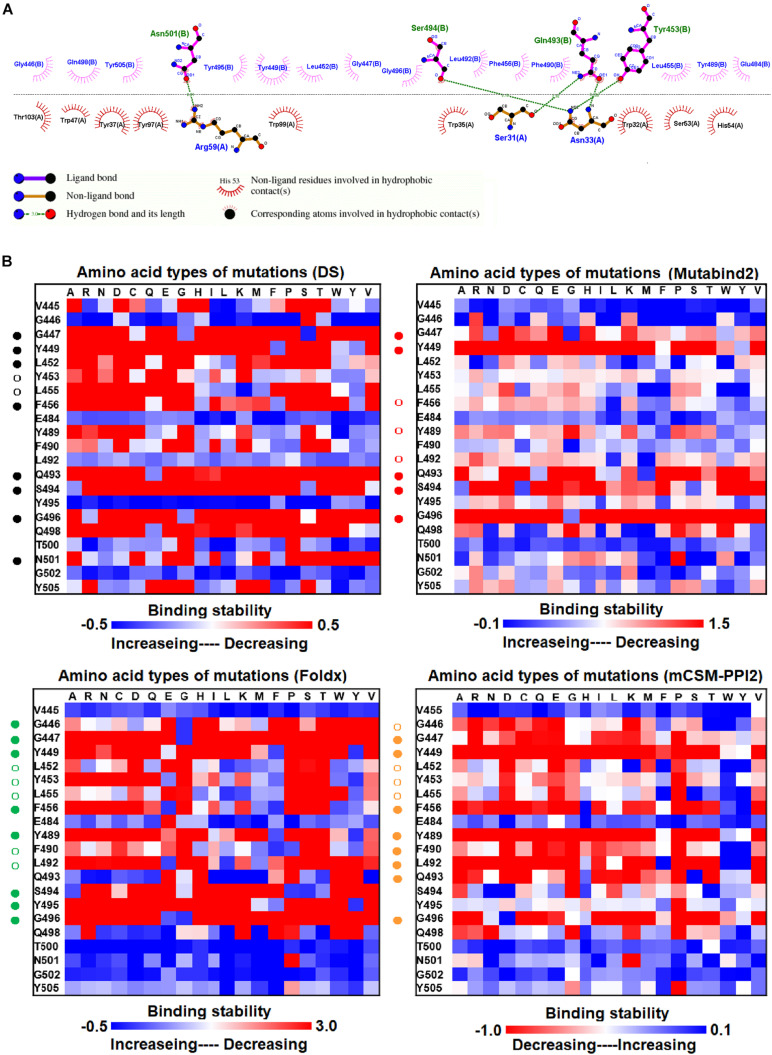
The mutational binding stabilities of RBD variants interacting to SR4 sybody. **(A)** The molecular interactions of SARS-CoV-2 RBD with SR4 sybody analyzed by ligplot. The chains A and B correspond to SR4 sybody and RBD, respectively. **(B)** The heatmap of interactive residues of RBD derived from the calculated mutational binding stabilities by using Discovery Studio 3.5 (DS), Mutabind2, FoldX, and mCSM-PPI2. The boxes of each mutations were colored with the gradient of a range between blue (stabilized binding) and red (destabilized binding). In all panels, the solid and hollow circles symbolize significant and moderate decreases of the binding stabilities, respectively.

We examined the destabilizing abilities of RBD variants in binding to SR2 and MR17 sybodies. The structure of the RBD–SR4 complex is illustrated in Figure 1C. Ligplot analyses demonstrate that the residues Y453, Q493, S494, and N501 of RBD interact with SR4 through hydrogen bond interactions (Figure 4A). Moreover, the residues G446, G447, Y449, L452, L455, F456, E484, Y489, F490, L492, Y495, G496, Q498, and Y505 interact with SR4 through hydrophobic contacts. Consequently, these interactive residues contributing to SR4 binding were used for mutational binding stability calculations. The results revealed that the residues G447, Y449, L452, L455, F456, Y489, F490, L492, Q493, S494, and G496 were mostly unfavorable for binding to SR4 when substituted by other amino acids (Figure 4B and Table 1).

**FIGURE 5:**
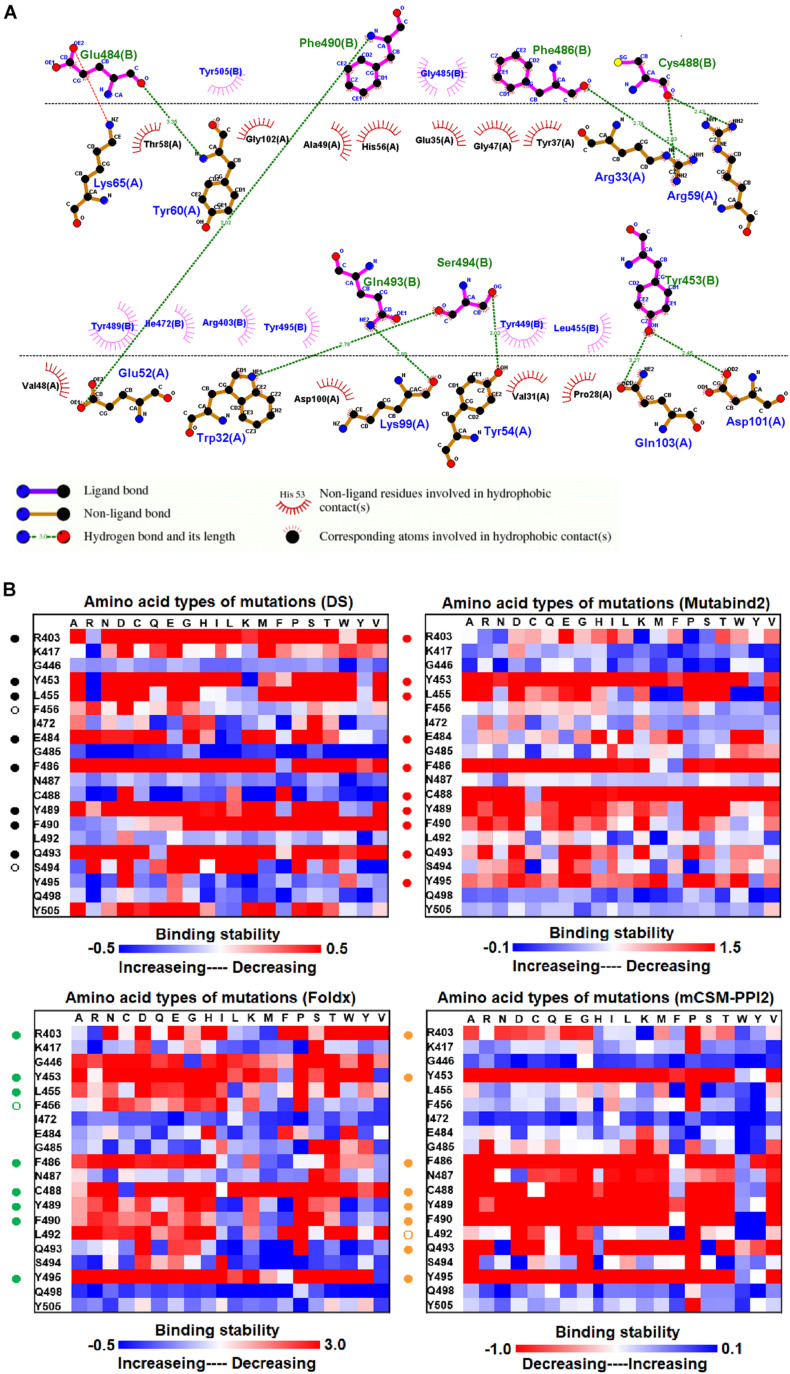
The mutational binding stabilities of RBD variants interacting to MR17 sybody. **(A)** The molecular interactions of SARS-CoV-2 RBD with MR17 sybody analyzed by ligplot. The chains A and B correspond to MR17 sybody and RBD, respectively. **(B)** The heatmap of interactive residues of RBD derived from the calculated mutational binding stabilities by using Discovery Studio 3.5 (DS), Mutabind2, FoldX, and mCSM-PPI2. The boxes of each mutations were colored with the gradient of a range between blue (stabilized binding) and red (destabilized binding). In all panels, the solid and hollow circles represent significant and moderate decreases of the binding stabilities, respectively.

The structure of the MR17 sybody, a LIama-derived single-domain antibody, in complex with RBD is illustrated in Figure 1D. MR17 engages in RBD at the receptor-binding motif (RBM). We analyzed molecular interactions between MR17 and RBD in detail (Figure 5A). The results revealed that RBD contacts MR17 through hydrogen bonding (Y453, E484, F486, C488, F490, Q493, and S494) and hydrophobic interactions (R403, L455, I472, G485, Y489, Y495, Y499, and Y505). These interactive residues of RBD were further mutated to other amino acid types to evaluate their effects on binding stability toward MR17. As presented in Figure 5B and Table 1, most of the mutations within the interactive residues R403, Y453, L455, E484, F486, C488, Y489, F490, Q493, and Y495 caused apparently unfavorable binding to MR17.

### Changes in the Binding Stability of RBD Variants Targeting P2B-2F6 Fab

**FIGURE 6:**
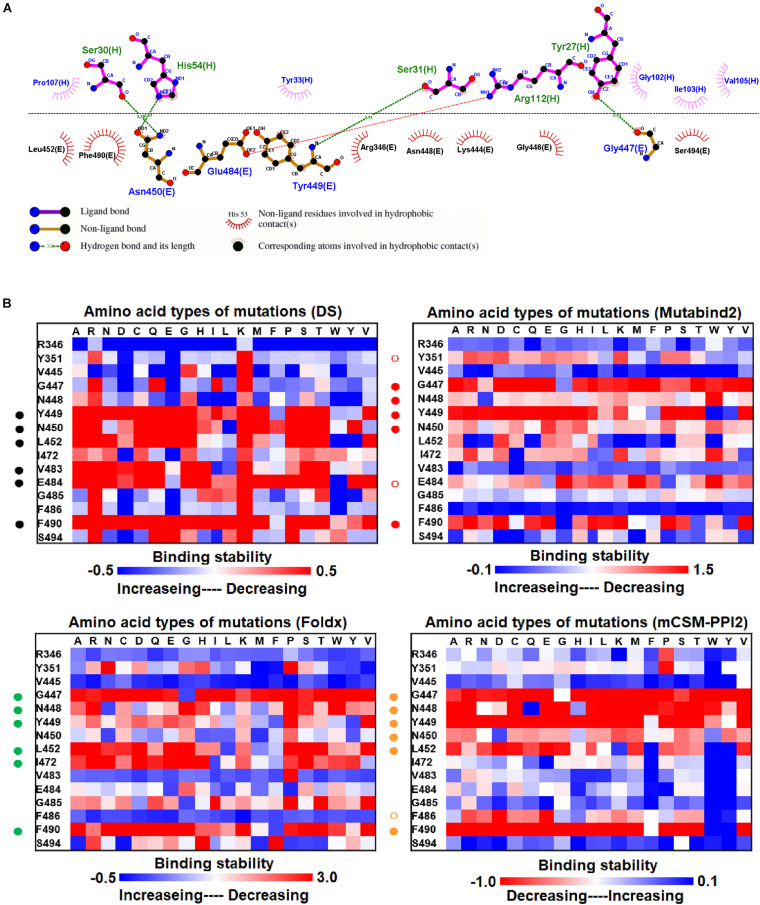
The mutational binding stabilities of RBD variants interacting to P2B-2F6 Fab. **(A)** The molecular interactions of SARS-CoV-2 RBD with P2B-2F6 Fab analyzed by ligplot. The chains E and H correspond to RBD and P2B-2F6 Fab sybody, respectively. **(B)** The heatmap of interactive residues of RBD derived from the calculated mutational binding stabilities by using Discovery Studio 3.5 (DS), Mutabind2, FoldX, and mCSM-PPI2. The boxes of each mutations were colored with the gradient of a range between blue (stabilized binding) and red (destabilized binding). In all panels, the solid and hollow circles denote significant and moderate decreases of the binding stabilities, respectively.

The mutational effects on the interactions of SARS-CoV-2 RBD variants with the plasma antibody P2B-2F6 Fab were also examined. The complex structure of P2B-2F6 Fab and SARS-CoV-2 RBD is illustrated in Figure 1B. P2B-2F6 Fab primarily interacts with RBD through its heavy chain. As illustrated in the ligplot (Figure 6A), the epitope residues are in the RBM of RBD, including Y449, N450, and E484 and K444, G446, G447, N448, L452, V483, G485, F490, and S494. We systematically analyzed their mutational effects on the binding stability of RBD toward P2B-2F6 Fab. The results revealed that the amino acid replacements of the interactive residues G447, Y449, N450, L452, V483, E484, and F490 resulted in unstable binding between RBD and P2B-2F6 Fab (Figure 6B and Table 1).

## Discussion

Epidemics have been affecting humans for decades (Khan et al., 2020). Millions of people have died from epidemics, including the 6th and 7th “EI Tor” cholera (Zhang et al., 2014), influenza A (H2N2) (Joseph et al., 2015), and HIV/AIDS (Gayle and Hill, 2001). At present, the COVID-19 pandemic presents a major global threat. Its causative agent, SARS-CoV-2, is an RNA virus and can thus mutate and evolve (Zheng, 2020). RNA viruses have higher mutation rates than DNA viruses (Sanjuan and Domingo-Calap, 2016). SARS-CoV-2 has been evolving at a rate of one–two mutations every month in the current pandemic (Callaway, 2020). The RBD domain of the S protein of SARS-CoV-2 directly binds to ACE2 to enter the host cell (Lan et al., 2020). Thus, RBD determines the transmissibility and infectivity of the virus, and all vaccines under development directly target it. Moreover, neutralizing antibodies have been developed against SARS-CoV-2 (Jiang et al., 2020; Pinto et al., 2020; Rogers et al., 2020; Xiaojie et al., 2020; Lau et al., 2021; Liu et al., 2021; Lu et al., 2021). However, RBD mutations can enable escape from the neutralizing immune response (Eguia et al., 2021; Greaney et al., 2021a). Thus, it is crucial to predict RBD mutations that can destabilize binding with neutralizing antibodies and weaken their effect.

Here, we comprehensively and systematically investigated the variants of RBD in terms of their binding stability to the antibodies. RBD residues that contact antibodies within a maximum distance of 5 Å from the antibodies’ interface were all considered to be interactive and substituted by all 20 amino acids for binding stability calculations. First, we found that variants of the RBD residues Y449, N450, L452, E484, Y489, F490, P491, L492, Q493, and S494 significantly reduced binding stability with the H11-D4 nanobody (Figure 2B and Supplementary Tables 1, 7, 13, 19). Notably, the residues N450, E484, F490, Q493, and S494 mainly interacted with the H11-D4 nanobody through hydrogen bonds; Y449, L452, Y489, and L492 contact the nanobody through hydrophobic interactions. Furthermore, the binding stability of RBD to the VH1-2-15 nanobody was disrupted when the residues Y449, E484, and S494 (primarily hydrogen bonds) and F456, G485, C488, Y489, F490, L492, and G496 (hydrophobic contacts) were subjected to single-amino acid mutations (Figure 3B and Supplementary Tables 2, 8, 14, 20). Similarly, the single-amino acid mutations of RBD also weaken its binding with the SR4 sybody (Figure 4B and Supplementary Tables 3, 9, 15, 21), in which the residues G447, Y449, L452, L455, F456, Y489, F490, L492, and G496 were connected by hydrophobic interactions, whereas residues Q493 and S494 were mostly connected through hydrogen bonds. In addition, the amino acid replacements impaired RBD binding to the MR17 sybody. Mutations at Y453, E484, F486, C488, F490, and Q493 (predominantly hydrogen bonds) and R403, L455, Y489, and Y495 (hydrophobic contacts) considerably destabilized binding stability (Figure 5B and Supplementary Tables 4, 10, 16, 22). Moreover, the variants causing apparent decreases in the binding stability of RBD to P2B-2F6 Fab were those with mutations at G447, Y449, N450, E484 (essentially hydrogen bonds), L452, V483, and F490 (mostly hydrophobic interactions) (Figure 6B and Supplementary Tables 5, 11, 17, 23). Additionally, we estimated the mutational effects on binding between RBD and ACE2 (Figure 1E and Supplementary Figure 1). The RBD residues K417, Y449, N487, and G502 interacted with ACE2 primarily through hydrogen bonding and were not favorable for binding when mutated to other amino acids (Supplementary Figure 1B and Supplementary Tables 6, 12, 18, 24). As well, the residues L455, F456, F486, Y489, Q498, N501, and Y505 in contact with ACE2 by hydrophobic interactions were prone to destabilize the binding after single-amino acid replacements. We therefore compared the variants that significantly detracted from the binding stability of RBD among all antibodies. We found that RBD variants mutated at R403, K417, G447, N448, Y449, N450, L452, Y453, L455, F456, E484, G485, F486, Y489, F490, Q493, S494, Y495, and G496 were unfavorable to binding with antibodies (Table 1). Notably, most of these residues were hydrophobic and aromatic, except for R403, K417, N448, N450, E484, Q493, and S494. Moreover, mutations at Y449, L452, L455, E484, Y489, F490, L492, Q493, and S494, which destabilize the binding, were concurrent with the high frequency observed in most antibodies in this study. These results imply that the residues Y449, L452, L455, E484, Y489, F490, L492, Q493, and S494 can be immune-escaping hotspots that may destabilize binding with antibodies and erode neutralizing immune responses.

Several experimental studies made effort to investigate the mutational escape from neutralizing and convalescent antibodies (Andreano et al., 2020; Li et al., 2020; Starr et al., 2020; Weisblum et al., 2020a; Garcia-Beltran et al., 2021a; Greaney et al., 2021a,b). Weisblum et al. (2020b) employed a recombinant chimeric VSV/SARS-CoV-2 reporter virus to investigate the mutations in the RBD, which confer resistance to monoclonal antibodies or convalescent plasma. They found E484K, F490L, and Q493K/R occured at high freequency during recombinant chimeric VSV/SARS-CoV-2 passage in the presence of neutralizing antibodies or plasma. In addition, the mutants E484K and Q493R caused apparently complete resistance to monoclonal antibodies (Weisblum et al., 2020b). Li et al. (2020) investigated 80 RBD variants for the infectivity and reactivity to a panel of neutralizing antibodies and sera from convalescent patients. They reported that most variants were less infectious, but L452R and F490L became resistant to some neutralizing antibodies (Li et al., 2020). Also, the immune escape of lineage B.1.351 of South Africa was examined and revealed that variants K417N/T, E484K, and N501Y were highly resistant to neutralization (Garcia-Beltran et al., 2021b). Greaney et al. (2021a) have mapped all the mutations to the RBD that escape binding by antibodies isolated from convalescent plasma. Their yeast-display deep mutational scanning revealed that antibodies were escaped by mutations to sites K417, N450, L452, L455, F456, E484, F486, F490, and Q493 (Greaney et al., 2021a). In our study, we analyzed that RBD residues Y449, L452, L455, E484, Y489, F490, L492, Q493, and S494 were hotspots with significantly destabilizing effects on binding to neutralizing antibodies. Especially, the identified hotspots, L452, L455, E484, F490, and Q493 are well consistent with the reported immune escape mutations from neutralizing and convalescent antibodies. Moreover, our findings that impaired binding stability of RBD and antibodies resulted from the mutational effects at L455, F456, G486, F486, and F490 (Table 1) are in good concordance with those of Greaney et al. (2021a) – mutations at sites near the structurally adjacent site of RBD’s receptor-binding ridge (e.g., L455, F456, G485, F486, and F490) have substantial antigenic effects. All these consistencies indicate the precision and reliance of our computational study in revealing the hotspots of SARS-CoV-2 RBD-specific neutralizing antibodies. In addition to the antibody immune escape of RBD, its binding and interaction to ACE2 were also characterized by *in vitro* and *in silico* mutational studies. Starr et al. (2020) systematically changed every amino acid in the RBD of the SARS-CoV-2 spike protein and determined the effects of the substitutions on RBD expression, folding, and ACE2 binding. They found that there are handful of sites where ACE2 binding imposes strong constrain (e.g., Y489, G502, and Y505), and mutations at interface residues (Y449, L455, F486, and Y505) enhance RBD expression but destabilize the effect of surface-exposed hydrophobic patches required for ACE2 binding. Mutations that enhance ACE2 binding affinity of RBD are notable at sites Q493, Q948, and N501. Besides, Teng et al. (2021) have conducted a computational study to investigate the effects of mutations on SARS-CoV-2 RBD-ACE2 binding affinity. They reported that mutations on residues G476, V483, Q498, T500, G496, and G502 exert apparently destabilizing impacts in RBD–ACE2 complex. In our study, we also analyzed the mutational effects of RBD on interacting with ACE2 (Supplementary Figure 1), in which variants of Y449, L455, F456, F486, N501, G502, and Y505 conspicuously impaired the binding stability, corroborating with Starr’s and Teng’s findings.

To verify our finding of possible immune-escape hotspots (Y449, L452, L455, E484, Y489, F490, L492, Q493, and S494), we compared our data with the current identified SARS-CoV-2 variants. Notably, the mutant E484K was detected in several SARS-CoV-2 variants (lineages B.1.525, B.1.526, B.1.1.7, B.1.351, P.1, and P2) (Table 1). E484 variants also abolished neutralization by monoclonal antibodies (Weisblum et al., 2020a; Chen et al., 2021). E484Q variant was detected in India (lineages B.1.617, B.1.617.1, and B.1.617.3). Consistently, the mutations at E484 considerably impaired RBD binding to antibodies in our study. Also, we observed that L452 variants destabilize RBD binding to antibodies in our prediction. Comparably, the variant L452R found in India (lineages B.1.617, B.1.617.1, B.1.617.2, and B.1.617.3) has not yet been demonstrated to become more infectious; however, it is becoming increasingly common in the United States (lineages B.1.427, B.1.429, and B.1.526.1). Furthermore, the SARS-CoV-2 variants F490S and S494P were identified as well in Peru (lineage C.37) and United States (lineage B.1.1.7), respectively. Similarly, we analyzed that these two variants exerted a destabilizing effect in the interactions of RBD and neutralizing antibodies as well. It is noteworthy that N501 was found to mutate to Y501 (N501Y) in the strain of the B1.1.7 lineage (Singh et al., 2021; Tang et al., 2021)^2^. This strain may be more transmissible and lethal and may be linked to a higher chance of hospitalization (Leung et al., 2021). In South Africa, in October 2020, the N501Y mutant was also detected in the strain of the B.1.351 lineage (Tegally et al., 2021). Also, variants K417N was found in Brazil (lineage P.1) and South Africa (lineage B.1.351), and K417T was identified in Japan (lineage P.1) (Table 1). Comparably, we found that K417N/T conspicuously disrupted the binding of RBD to M17 sybody, although only in the prediction by DS. Our predictions also observed that N501Y showed apparently increased binding stability in the complex of RBD–ACE2, corroborating with the current isolated variant (N501Y). These reports strongly support the hotspots (L452, L455, F456, E484, F486, F490, and S494) that we found and indicate that our findings are precise and reliable for further use in antibody engineering or vaccine developments.

**FIGURE 7:**
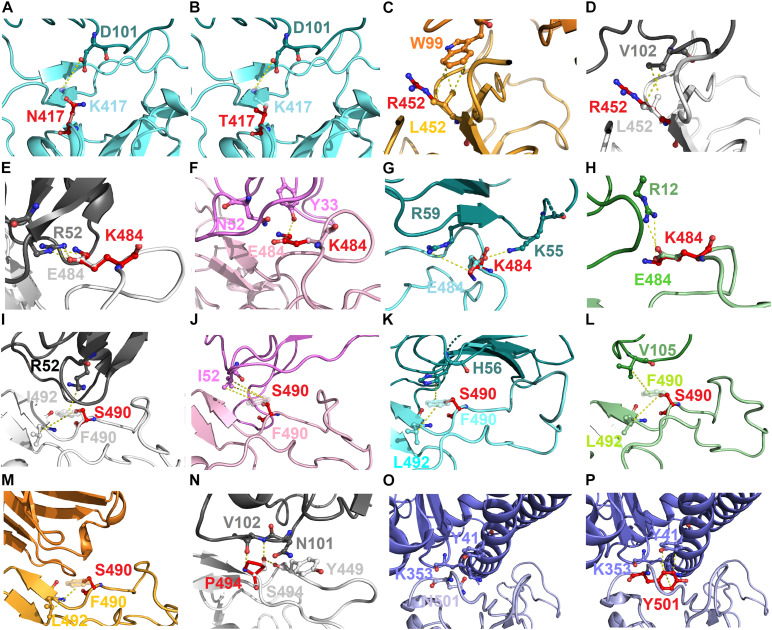
The molecular interactions of SARS-CoV-2 RBD variants identified from pandemic isolates. **(A)** The compared intermolecular interactions of K417 (light cyan stick) and its variant N417 (red stick) in the complex of RBD–MR17. **(B)** The overlapped view of intermolecular interactions of K417 (light cyan stick) and its variant N417 (red stick) in the complex of RBD–MR17. **(C)** The compared intermolecular interactions of L452 (light orange stick) and its variant R452 (red stick) in the complex of RBD–SR4. **(D)** The overlapped view of intermolecular interactions of L452 (light gray stick) and its variant R452 (red stick) in the complex of RBD–H11-D4. **(E)** The compared intermolecular interactions of E484 (light gray stick) and its variant K484 (red stick) in the complex of RBD–H11-D4. **(F)** The overlapped view of intermolecular interactions of E484 (light pink stick) and its variant K484 (red stick) in the complex of RBD–VH1-2-15. **(G)** The intermolecular interactions of E484 (light cyan stick) and its variant K484 (red stick) in the complex of RBD–MR17. **(H)** The intermolecular interactions of E484 (light green stick) and its variant K484 (red stick) in the complex of RBD–P2B-2F6. **(I)** The compared intermolecular interactions of F490 (light gray stick) and its variant S490 (red stick) in the complex of H11–D4. **(J)** The overlapped view of intermolecular interactions of F490 (light pink stick) and its variant S490 (red stick) in the complex of RBD–VH1-2-15. **(K)** The intermolecular interactions of F490 (light cyan stick) and its variant S490 (red stick) in the complex of RBD–MR17. **(L)** The intermolecular interactions of F490 (light green stick) and its variant S490 (red stick) in the complex of RBD–P2B-2F6. **(M)** The intermolecular interactions of F490 (light orange stick) and its variant S490 (red stick) in the complex of RBD–SR4. **(N)** The intermolecular interactions of S494 (light gray stick) and its variant P494 (red stick) in the complex of RBD–H11-D4. **(O)** The intermolecular interactions of N501 (light purple stick) in the complex of RBD–ACE2. **(P)** The intermolecular interactions of Y501 (red stick) in the complex of RBD–ACE2. In all panels, proteins are shown in ribbons and the molecular interactions are presented as dash lines.

To explore the possible mechanism of action of the identified hotspots, we further investigated the molecular interactions of mutational variations in the structural complex of RBD–antibody. The residue K417 of RBD connected with D101 of MR17 by charge–charge interactions. Thus, RBD–MR17 complex could be destabilized when K417 was mutated to non-charged N417 and T417 (Figures 7A,B). Structurally, the residue L452 of RBD contacted with W99 and V102 of SR-4 and H11-D4, respectively, through hydrophobic interactions. These hydrophobic contacts are probably disrupted as L452 is substituted by an arginine residue, causing the decreased binding affinity of RBD with SR-4 and H11-D4 (Figures 7C,D). In addition, the charge–charge interactions can also be seen in residue E484, which interacts with R52, R59, and R12 of H11-D4, MR17, P2B-2F6, individually. As well, E484 formed hydrogen bond with Y33 of VH1-2-15. All these molecular interactions were abolished while E484 was replaced by a lysine residue. This could also detract from the binding affinity of RBD with neutralizing antibodies (Figures 7E–H). Moreover, the aromatic residue F490 of RBD makes contributions in binding to antibodies by exerting hydrophobic and cation–pi interactions (Figures 7I–M). These molecular interactions were disrupted when F490 was mutated to S490, therefore destabilizing the binding stability of RBD and antibodies. Notably, the mutation S494P could breakdown the hydrogen bond interactions of S494 (RBD) with N101 and V102 of H11-D4, thus weakening the binding affinity (Figure 7N). The variant N501Y showed extra hydrophobic interactions with Y41 and K353, significantly increasing the binding stability of RBD to ACE2 (Figures 7O,P), explaining the high concurrency of the N501Y variant in several countries. Taken together, our data revealed all the possible hotspots that may substantially impair the binding of SARS-CoV-2 RBD to both ACE2 and neutralizing antibodies. Our findings will benefit the development and engineering of new and potent antibodies and vaccines against SARS-CoV-2.

## Conclusion

In this study, we explored the possible hotspots of SARS-CoV-2 RBD that can enable virus escape from recognition by neutralizing antibodies. Computational analyses demonstrated that specific variants of RBD significantly impair binding to neutralizing antibodies. Particularly, the RBD residues Y449, L452, L455, E484, Y489, F490, L492, Q493, and S494 were found to be hotspots because their variants could markedly destabilize the binding to neutralizing antibodies. Notably, the hotspots K417, L452, L455, E484, F490, and S494 were supported by evidence from the literature. The hotspots Y449, L455, and Y489 were commonly observed to disrupt the binding to ACE2 and neutralizing antibodies. Conclusively, our data provide insights into the putative impacts of the possible immune-escaping hotspots on interactions with neutralizing antibodies, which can help develop new therapeutic agents against potential variants of SARS-CoV-2.

## Data Availability Statement

The original contributions presented in the study are included in the article/Supplementary Material, further inquiries can be directed to the corresponding author.

## Author Contributions

T-ST and K-CT designed the experiments and wrote the manuscript. T-ST, K-CT, and Y-CL performed the experiments and analyzed the data. All authors contributed to the article and have approved the submitted version.

## Conflict of Interest

The authors declare that the research was conducted in the absence of any commercial or financial relationships that could be construed as a potential conflict of interest.

## Footnotes

1 https://www.worldometers.info/coronavirus/

2 cov-lineages.org
