# Supplementary material for: Comprehensive Deep Mutational Scanning Reveals the Immune-Escaping Hotspots of SARS-CoV-2 Receptor-Binding Domain Targeting Neutralizing Antibodies
Source: Front Microbiol. 2021 Jul 15;12:698365. doi: 10.3389/fmicb.2021.698365 (PMC8319916; doi:10.3389/fmicb.2021.698365)
Supplement: Supplementary file 1 [file Data_Sheet_1.docx]

Comprehensive deep mutational scanning reveals the immune-escaping hotspots of SARS-CoV-2 receptor-binding domain targeting neutralizing antibodies

Keng-Chang Tsai ^1,2^, Yu-Ching Lee^2,3,4,5,6^ Tien-Sheng Tseng^7*^

^1^ National Research Institute of Chinese Medicine, Ministry of Health and Welfare, Taipei, Taiwan.

^2^ Ph.D. Program in Medical Biotechnology, College of Medical Science and Technology, Taipei Medical University, Taipei, Taiwan.

^3^TMU Research Center of Cancer Translational Medicine, Taipei Medical University, Taipei, Taiwan.

^4^Ph.D. Program for Cancer Molecular Biology and Drug Discovery, College of Medical Science and Technology, Taipei Medical University, Taipei, Taiwan.

^5^Ph.D. Program in Biotechnology Research and Development, College of Pharmacy, Taipei Medical University, Taipei, Taiwan.

^6^Biomedical Commercialization Center, Taipei Medical University, Taipei, Taiwan.

^7^ Institute of Molecular Biology, National Chung Hsing University, Taichung, Taiwan.

*** Correspondence:**Tien-Sheng Tseng
emersontseng@dragon.nchu.edu

Keywords: SARS-CoV-2, COVID-19, Binding Stability, Hotspots, Neutralization, Antibody, Immunity.

**Supporting information**

**Figures**

**
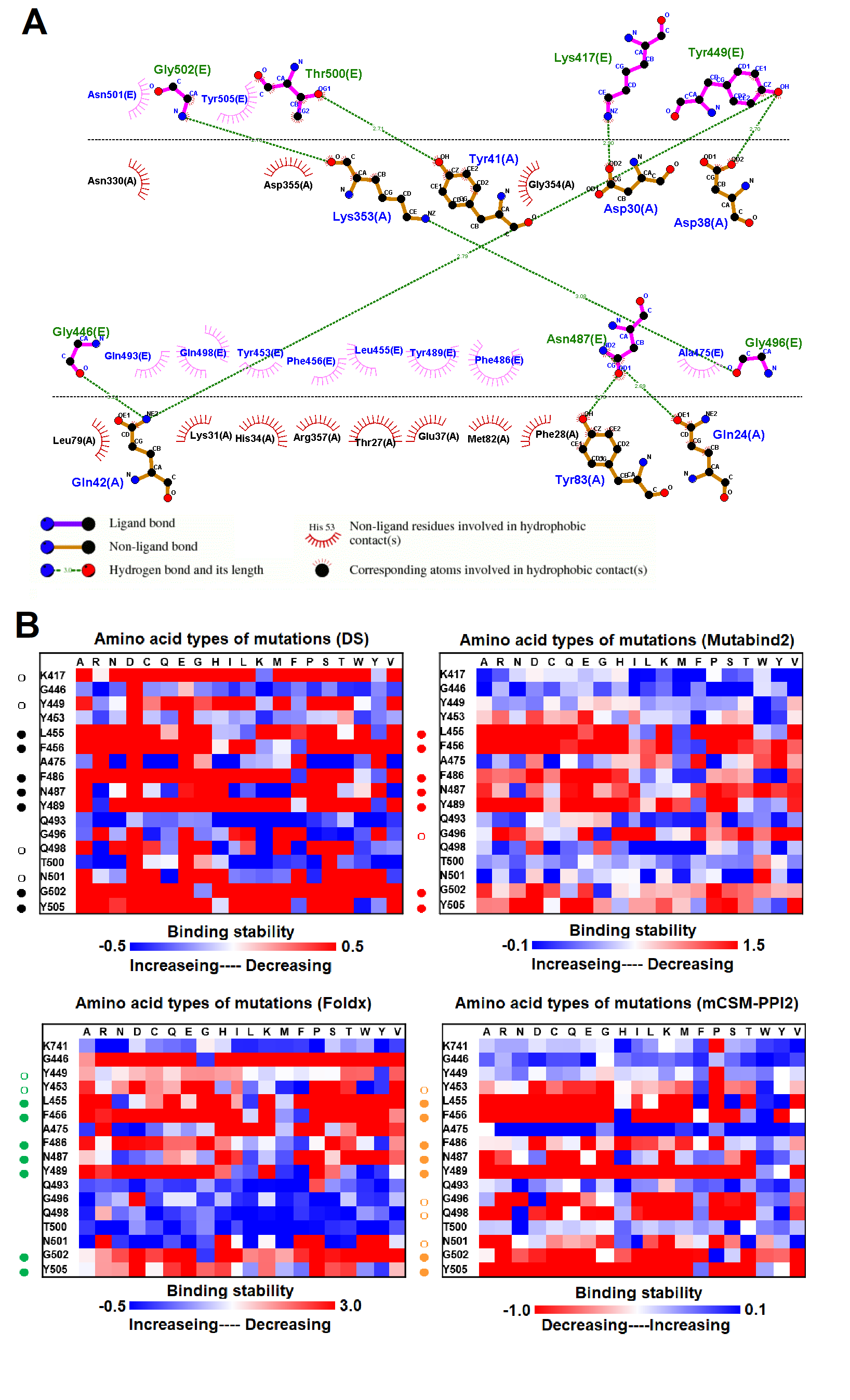
**

**Figure S1. The mutational binding stabilities of RBD variants interacting to ACE2.** (**A**) The molecular interactions of SARS-CoV-2 RBD with ACE2 analyzed by ligplot. The chain A and E correspond to ACE2 and RBD, respectively. (**B**) The heatmap of interactive residues of RBD derived from the calculated mutational binding stabilities by using Discovery Studio 3.5 (DS), Mutabind2, FoldX, and mCSM-PPI2. The boxes of each mutations were colored with the gradient of a range between blue (stabilized binding) and red (destabilized binding). In all panels, the solid and hollow circles denote significant and moderate decreases of the binding stabilities, respectively.

**Tables**

**Table S1. The predicted binding stabilities of SARS-CoV-2 RBD targeting H11-D4 by DS.**

|  | **A** | **R** | **N** | **D** | **C** | **Q** | **E** | **G** | **H** | **I** | **L** | **K** | **M** | **F** | **P** | **S** | **T** | **W** | **Y** | **V** |
| --- | --- | --- | --- | --- | --- | --- | --- | --- | --- | --- | --- | --- | --- | --- | --- | --- | --- | --- | --- | --- |
| **R346** | -0.41 | 0 | -0.43 | -0.93 | -0.41 | -0.43 | -0.97 | -0.4 | -0.42 | -0.45 | -0.44 | 0.32 | -0.45 | -0.65 | -0.42 | -0.4 | -0.43 | -0.71 | -0.39 | -0.44 |
| **K444** | -1.35 | -0.72 | -1.34 | -1.68 | -1.33 | -0.86 | -1.38 | -1.35 | -1.21 | -1.25 | -1.39 | -0.03 | -1.02 | -1.37 | -1.4 | -1.3 | -1.35 | -1.4 | -1.53 | -1.35 |
| **G446** | -0.05 | 0.05 | -0.09 | -0.28 | 0.02 | -0.04 | -0.09 | 0 | -0.52 | -0.4 | -0.53 | 0.46 | -0.01 | -0.72 | -0.06 | 0.35 | 0.11 | -1.49 | -0.69 | -0.23 |
| **Y449** | 1.67 | 2.08 | 0.82 | 1.14 | 1.34 | 1.31 | 1.58 | 2.54 | 0.62 | 2.22 | 0.55 | 1.82 | 1.45 | -0.03 | 0.76 | 1.93 | **2.76** | 0.09 | 0 | 0.46 |
| **N450** | 0.98 | 1.77 | 0 | 0.22 | 0.77 | 0.57 | 0.63 | 1.12 | 0.9 | 0.7 | 0.12 | 1.92 | 0.89 | -0.06 | 0.36 | 1.36 | 1.15 | -0.45 | -1.11 | 0.18 |
| **L452** | 0.97 | 1.25 | 0.48 | 0.45 | 0.62 | 0.24 | 0.24 | 1.03 | 0.26 | 0.17 | 0 | 1.54 | 0.34 | 0.26 | 0.59 | 0.67 | 0.22 | -0.22 | 0.17 | 0.41 |
| **L455** | 0.74 | 0.2 | 0.32 | 0.19 | 0.62 | -0.07 | -0.2 | 0.83 | -0.02 | 0.29 | -0.38 | 0.36 | 0.13 | -0.08 | 0.51 | 0.68 | 0.37 | 0.14 | -0.07 | 0.4 |
| **F456** | 0.25 | 0.38 | 0.18 | -0.17 | 0.17 | 0.03 | -0.16 | 0.27 | -0.22 | 0.1 | 0.01 | 0.26 | -0.25 | -0.07 | 0.07 | 0.22 | 0.19 | 1.44 | -0.04 | 0.15 |
| **T470** | -0.31 | 0.76 | -0.21 | -0.76 | -0.31 | 1.16 | -0.2 | -0.33 | -0.17 | 0.92 | -0.26 | 1.02 | 0.08 | -0.16 | -0.29 | -0.1 | -0.01 | -0.14 | -0.16 | -0.18 |
| **G482** | -0.07 | 0.41 | -0.12 | -0.69 | -0.14 | -0.33 | -0.77 | -0.03 | -0.11 | -0.32 | -0.17 | 0.51 | -0.11 | -0.31 | -0.18 | -0.11 | -0.13 | -0.18 | -0.17 | -0.26 |
| **V483** | 0.06 | 0.52 | -0.02 | -0.56 | 0.07 | 0 | -0.52 | 0.2 | -0.01 | -0.07 | -0.1 | 0.68 | 0.04 | -0.13 | 0.08 | 0.11 | -0.04 | -1.42 | -0.5 | -0.08 |
| **E484** | 1.81 | 2.18 | 1.53 | 0.77 | 1.69 | -0.07 | 0.01 | 2.56 | 0.72 | -0.03 | -0.07 | 2.15 | 1.65 | 2.17 | 4.12 | 2.11 | 1.06 | 1.82 | 2.53 | 1.17 |
| **Y489** | 0.46 | -0.01 | 0.06 | -0.01 | 0.27 | 0.14 | -0.07 | 0.5 | -0.2 | -0.48 | -0.39 | 1.02 | 0.31 | -0.01 | -0.06 | 0.45 | -0.67 | 0.11 | -0.01 | -0.81 |
| **F490** | 2.13 | 2.48 | 1.06 | 1.27 | 1.68 | 2.2 | 1.86 | 3.27 | 0.32 | 2.32 | 0.99 | 4.46 | 1.81 | -0.01 | 4.11 | 1.78 | 2.34 | 1.84 | -0.22 | 1.56 |
| **P491** | 0.14 | 0.71 | 0.2 | -0.14 | 0.15 | 0.17 | -0.25 | 0.14 | 0.15 | 0.18 | 0.18 | 0.61 | 0.07 | 0.1 | -0.01 | 0.19 | 0.2 | 0.17 | 0.09 | 0.08 |
| **L492** | 0.03 | 0.74 | 0.03 | -0.31 | -0.02 | -0.33 | -0.7 | 0.19 | -0.11 | -0.16 | 0 | 0.56 | -0.04 | 0.09 | -0.03 | -0.08 | 0.06 | -0.24 | -0.03 | -0.07 |
| **Q493** | 1.39 | 1.04 | 0.31 | 0.76 | 1.13 | -0.01 | -0.01 | 1.9 | 0.58 | 0.23 | -0.31 | 1.88 | 1.05 | 1.42 | 1.01 | 1.47 | 0.5 | 0.88 | 1.43 | 0.58 |
| **S494** | 0.03 | 3.3 | 2.17 | 1.91 | 0.26 | 0.19 | 0.28 | 0.94 | 1.06 | 2.87 | 1.66 | 1.97 | 1.01 | 5.18 | 11.97 | -0.01 | -0.04 | 7.55 | 4.48 | 0.9 |

**Table S2. The predicted binding stabilities of SARS-CoV-2 RBD targeting VH1-2-15 by DS.**

|  | **A** | **R** | **N** | **D** | **C** | **Q** | **E** | **G** | **H** | **I** | **L** | **K** | **M** | **F** | **P** | **S** | **T** | **W** | **Y** | **V** |
| --- | --- | --- | --- | --- | --- | --- | --- | --- | --- | --- | --- | --- | --- | --- | --- | --- | --- | --- | --- | --- |
| **G446** | -0.53 | -1.28 | -0.56 | -1.5 | -0.92 | -0.85 | -1.75 | -0.09 | -1.25 | -1.67 | -0.58 | -0.62 | -1.55 | -1.86 | -0.93 | 1.43 | -0.6 | -2.49 | -1.17 | -1.08 |
| **Y449** | 2.39 | 1.48 | 2.19 | 1.4 | 2.06 | 1.29 | 1.35 | 2.89 | 1.04 | 1.56 | 1.65 | 2.22 | 1.62 | 0.1 | 1.9 | 2.84 | 2.85 | 0.68 | -0.09 | 2.34 |
| **N450** | -0.26 | 0.17 | -0.1 | -0.74 | -0.28 | -0.43 | -0.76 | -0.23 | -0.13 | -0.39 | -0.3 | 0.18 | -0.54 | -0.24 | -0.42 | -0.27 | -0.37 | -0.44 | -0.15 | -0.32 |
| **L452** | 0.47 | 1.3 | 0.11 | 0.07 | 0.41 | 0.5 | 0.55 | 0.53 | 0.15 | 0.15 | -0.08 | 0.85 | 0.48 | 0.38 | 0.43 | 0.49 | 0.21 | 0.15 | 0.25 | 0.27 |
| **L455** | 0.42 | -0.4 | 0.22 | 0.01 | 0.33 | -0.01 | -0.06 | 0.48 | -0.08 | -0.07 | 0.02 | 0.3 | -0.14 | -0.57 | 0.24 | 0.37 | 0.37 | -0.17 | -0.82 | 0.36 |
| **F456** | 0.45 | 0.31 | 0.45 | 0.05 | 0.36 | 0.32 | 0.1 | 0.47 | 0.26 | 0.28 | 0.24 | 0.54 | 0.13 | -0.04 | 0.36 | 0.4 | 0.42 | 0.7 | -0.39 | 0.36 |
| **G482** | -0.05 | 0.34 | -0.05 | -0.33 | 0 | 0 | -0.35 | 0 | -0.02 | -0.01 | -0.05 | 0.34 | -0.05 | -0.04 | -0.02 | 0.02 | -0.07 | 0.05 | -0.02 | -0.11 |
| **V483** | 0.64 | -0.29 | 0.02 | 0.39 | 0.63 | 0.3 | 0.32 | 0.8 | 0.25 | -0.23 | 0.26 | 0.42 | -0.13 | -0.95 | 0.48 | 1.04 | 0.31 | -1.93 | -1.28 | 0 |
| **E484** | 0.72 | -0.27 | 0.18 | 0.91 | 0.31 | -0.11 | 0 | 0.78 | -0.02 | 0.64 | 0.67 | -0.14 | -0.36 | 0.6 | -0.12 | 0.5 | 0.29 | 1.89 | 0.42 | 0.31 |
| **G485** | 5 | 10 | 1.16 | 1.45 | 3.77 | 5.56 | 3.73 | 0 | 2.56 | 9.87 | 10 | 10 | 0.51 | 3.4 | 0.66 | 5.71 | 1.3 | 4.83 | 3.07 | 9.32 |
| **C488** | -0.05 | 0.2 | -0.33 | -0.19 | -0.07 | -0.36 | -0.32 | -0.19 | 2.72 | -0.4 | -0.4 | 0.17 | -0.37 | 4.6 | -0.51 | -0.14 | -0.14 | 1.71 | 1.21 | -0.16 |
| **Y489** | 0.51 | -0.11 | 0.47 | 0.31 | 0.46 | 0.51 | 0.47 | 0.59 | 0.33 | 0.16 | 0.3 | 1.34 | 0.34 | 0.3 | 0.35 | 0.53 | 0.63 | 0.28 | 0.4 | 0.18 |
| **F490** | 1.95 | 2.46 | 1.46 | 1.96 | 1.63 | 1.98 | 3.01 | 2.44 | 0.81 | 1.16 | 0.73 | 2.87 | 1.25 | 0 | 0.93 | 2.16 | 1.19 | -0.71 | -0.02 | 1.09 |
| **L492** | 0.12 | 0.6 | 0.13 | -0.04 | 0.08 | -0.41 | -0.3 | 0.31 | 0.03 | -0.09 | 0 | 0.34 | 0.01 | -0.03 | -0.1 | 0.09 | 0.05 | -0.05 | -0.06 | -0.25 |
| **Q493** | 0.44 | -1.59 | 0.03 | 0.01 | 0.38 | -0.05 | -0.03 | 0.6 | -0.51 | -0.34 | -1.01 | 0.06 | 0.2 | -0.69 | 0.29 | 0.68 | 0.04 | 0.34 | 0.17 | -0.24 |
| **S494** | -0.29 | -0.66 | -1.38 | -1.06 | -0.11 | 0.32 | -1.23 | 0.22 | -0.38 | -1.12 | 0.3 | 0.42 | -0.79 | 0.77 | 1.11 | -0.21 | -0.86 | 3.09 | 4.38 | -0.59 |
| **G496** | -0.09 | 1.51 | -0.23 | -0.37 | -0.2 | 0.03 | 0.28 | -0.11 | -1.04 | 1.1 | -1.87 | 2.17 | 1.1 | -1.14 | -0.84 | -0.13 | 0.25 | -1.18 | -0.21 | -0.69 |
| **Q498** | 0 | 1.07 | 0.09 | -0.71 | 0.11 | -0.07 | -0.71 | 0.13 | 0.04 | -0.12 | -0.05 | 0.95 | -0.81 | -0.09 | -0.17 | 0.03 | -0.13 | -0.34 | -0.08 | -0.13 |

**Table S3. The predicted binding stabilities of SARS-CoV-2 RBD targeting SR4 by DS.**

|  | **A** | **R** | **N** | **D** | **C** | **Q** | **E** | **G** | **H** | **I** | **L** | **K** | **M** | **F** | **P** | **S** | **T** | **W** | **Y** | **V** |
| --- | --- | --- | --- | --- | --- | --- | --- | --- | --- | --- | --- | --- | --- | --- | --- | --- | --- | --- | --- | --- |
| **V445** | 0.73 | -0.28 | 0.27 | 0.68 | 0.46 | -0.13 | 0.27 | 0.93 | 0.61 | -0.46 | -0.54 | -0.13 | 0.24 | 0.44 | 0.62 | 1.14 | 0.5 | 0.11 | 0.4 | -0.01 |
| **G446** | -0.39 | -1.21 | -0.52 | 0.29 | -0.41 | -0.68 | -0.4 | -0.05 | 0.24 | -1.15 | -0.91 | -0.36 | -0.83 | -1.59 | -1.07 | 0.68 | 0.15 | -0.68 | -1.05 | -1.32 |
| **G447** | 0.59 | 7.55 | 0.71 | 2.79 | 0.32 | 2.97 | 4.62 | -0.04 | 2.48 | 5.21 | 3.69 | 5.1 | 8.34 | 6.57 | 2.71 | -0.4 | 0.86 | 6.72 | 7.21 | 1.35 |
| **Y449** | 2.04 | 1.64 | 1.51 | 2.19 | 1.95 | 2.4 | 2.52 | 2.53 | 0.76 | 1.13 | 1.11 | 0.85 | 0.88 | -0.11 | 1.25 | 2.46 | 2.64 | 0.23 | 0 | 1.92 |
| **L452** | 0.77 | 0.63 | 0.45 | 1.03 | 0.69 | 0.36 | 0.64 | 0.8 | 0.41 | 0.23 | -0.01 | 1.31 | 0.48 | 0.76 | 0.58 | 0.79 | 0.52 | 0.2 | 0.43 | 0.44 |
| **Y453** | 0.47 | 0.57 | 0.46 | 0.63 | 0.36 | 0.6 | 0.59 | 0.42 | 0.19 | 0.47 | 0.33 | 0.58 | 0.17 | 0.2 | 0.28 | 0.44 | 0.33 | -0.02 | -0.01 | 0.43 |
| **L455** | 1.14 | 1.82 | 0.53 | 1.35 | 1.03 | 1.14 | 2.39 | 1.31 | 0.27 | 0.03 | 0 | -1.22 | -0.01 | 0.7 | 0.78 | 1.13 | 0.9 | 0.41 | -0.07 | 0.75 |
| **F456** | 0.81 | 1.41 | 0.53 | 0.8 | 0.68 | 0.37 | 0.67 | 0.81 | 0.12 | 0.73 | 0.46 | 0.47 | 0.48 | 0 | 0.55 | 0.79 | 0.76 | 2.1 | 0.06 | 0.68 |
| **E484** | -0.12 | -0.22 | -0.21 | -0.21 | -0.14 | -0.03 | 0 | -0.04 | -0.99 | -0.37 | -0.81 | 0.06 | -0.99 | -1.58 | -0.2 | -0.12 | -0.15 | -0.48 | -0.38 | -0.29 |
| **Y489** | 0.58 | 0.48 | 1.07 | 0.59 | 0.54 | 0.13 | 0.35 | 0.78 | 0.09 | 0.27 | 0.41 | 0.48 | 0.07 | 0 | 0.2 | 0.51 | 0.39 | -0.54 | 0 | 0.07 |
| **F490** | 0.45 | 0.46 | 0.19 | 0.5 | 0.3 | 0.35 | 0.72 | 0.67 | 0.11 | 1.23 | 0.07 | 1.53 | 0.37 | -0.07 | -0.11 | 0.65 | 1.03 | 0.36 | -0.07 | 0.12 |
| **L492** | -0.01 | -0.07 | 0.06 | 0.14 | -0.03 | -0.13 | -0.01 | 0.15 | -0.03 | -0.17 | -0.01 | 0.02 | -0.07 | 0.12 | 0.34 | -0.09 | -0.2 | -0.2 | 0.01 | -0.09 |
| **Q493** | 2.32 | 1.18 | 1.39 | 2.9 | 2.16 | -0.01 | 2.29 | 2.7 | 0.49 | 0.48 | 1.64 | 1.24 | 2.08 | 2.55 | 1.67 | 2.28 | 2.3 | 0.54 | 1.7 | 1.35 |
| **S494** | 0.02 | 3.06 | 1.48 | 2.98 | 0.6 | 5.06 | 6.14 | 0.77 | 4.87 | 2.83 | 4.57 | 5.01 | 3.88 | 7.15 | 3.62 | 0 | 0.95 | 0.75 | 9.02 | 0.34 |
| **Y495** | -0.52 | -0.37 | -0.52 | -0.43 | -0.59 | -0.58 | -0.46 | -0.6 | -0.48 | -0.47 | -0.47 | -0.61 | -0.57 | 0.05 | -0.76 | -0.56 | -0.57 | 0.06 | 0 | -0.8 |
| **G496** | 1.13 | 0.16 | 0.79 | 2.94 | 0.93 | 2.91 | 4.72 | 0 | 2.75 | 2.04 | 3.5 | 4.9 | 3.41 | 1.33 | 1.33 | 0.41 | 1.92 | 5.16 | 3.23 | 2.11 |
| **Q498** | 1.25 | 3.85 | 0.53 | 2.13 | 0.7 | 0 | 1.13 | 1.74 | 0.49 | 1.89 | 0.73 | 0.48 | 1.75 | 0.58 | 0.66 | 1.52 | 0.72 | 3.27 | 0.39 | 0.24 |
| **T500** | 0.2 | -0.87 | -0.09 | 0.13 | 0.04 | 0.02 | 0.26 | 0.53 | -0.16 | -1.15 | -1.43 | 0.09 | 0.02 | -0.21 | 3.33 | 0.13 | 0 | -0.63 | -0.18 | -0.43 |
| **N501** | 0.87 | 0.29 | 0 | 0.26 | 0.52 | 0.25 | 0.68 | 1.06 | 0.08 | 0.72 | -0.23 | 0.42 | 0.57 | 0.08 | 2.72 | 0.75 | 0.67 | 1.43 | 1.32 | 1.22 |
| **G502** | -0.1 | -0.46 | -0.31 | -0.41 | -0.1 | -0.09 | -0.41 | 0 | -0.49 | -0.39 | -0.79 | -0.12 | -0.28 | -1.21 | -0.99 | -0.08 | -0.16 | -0.44 | -0.59 | -0.12 |
| **Y505** | 0.32 | 0.97 | 0.02 | -0.02 | 0.15 | 1.32 | 0.96 | 0.63 | -0.09 | 0.07 | 0.22 | 1.78 | 0.92 | -0.31 | 0 | 0.78 | 0.23 | -0.47 | -0.01 | -0.1 |

**Table S4. The predicted binding stabilities of SARS-CoV-2 RBD targeting MR17 by DS.**

|  | **A** | **R** | **N** | **D** | **C** | **Q** | **E** | **G** | **H** | **I** | **L** | **K** | **M** | **F** | **P** | **S** | **T** | **W** | **Y** | **V** |
| --- | --- | --- | --- | --- | --- | --- | --- | --- | --- | --- | --- | --- | --- | --- | --- | --- | --- | --- | --- | --- |
| **R403** | 0.92 | 0.01 | 0.98 | 1.65 | 0.93 | 0.9 | 1.93 | 0.89 | 1.49 | 0.93 | 0.64 | 0.45 | 1.21 | 0.76 | 0.89 | 0.96 | 0.91 | 0.27 | 1.45 | 0.87 |
| **K417** | 0.23 | -0.95 | 0.32 | 0.86 | 0.22 | 0.43 | 0.98 | 0.24 | 0.23 | 0.4 | 0.22 | 0 | 0.62 | 0.36 | 0.28 | 0.27 | 0.35 | 0.44 | 0.39 | 0.33 |
| **G446** | -0.03 | -0.09 | -0.06 | 0.05 | -0.08 | -0.11 | 0.04 | 0 | -0.2 | -0.2 | -0.21 | -0.09 | -0.06 | -0.08 | -0.07 | -0.01 | -0.09 | -0.49 | -0.08 | -0.24 |
| **Y453** | 1.02 | -0.71 | 1.06 | 1.89 | 0.97 | 1.01 | 2.03 | 0.96 | 0.62 | 1.1 | 0.93 | -0.63 | 0.41 | 0.29 | 0.93 | 1.01 | 1.25 | 1.24 | 0 | 1.27 |
| **L455** | 1.21 | -1.24 | 0.82 | 1.85 | 1.12 | 0.15 | 1.44 | 1.35 | 0.22 | 0.21 | 0 | -0.04 | 0.63 | 0.53 | 1.4 | 1.24 | 0.96 | 0.58 | 0.13 | 0.93 |
| **F456** | 0.32 | 0.46 | 0.25 | 0.5 | 0.22 | 0.27 | 0.45 | 0.34 | 0.1 | 0.2 | 0.16 | 0.04 | -0.06 | 0 | 0.15 | 0.34 | 0.25 | 0.17 | 0.11 | 0.28 |
| **I472** | 0.37 | -0.13 | 0.29 | 0.09 | 0.28 | -0.42 | -0.2 | 0.46 | 0.44 | -0.37 | -0.31 | -0.06 | 0.15 | -0.68 | 0.28 | 0.61 | 0.31 | -0.95 | -0.03 | -0.03 |
| **E484** | 0.69 | 0.5 | 0.47 | 0.47 | 0.52 | 0.72 | -0.01 | 1.29 | 0.33 | -0.73 | -0.3 | 0.7 | 0.49 | 0.15 | 0.94 | 1.03 | 0.4 | 0.22 | 0.21 | -0.13 |
| **G485** | -0.25 | -0.56 | -0.86 | -0.67 | -0.39 | -0.37 | -0.34 | -0.02 | -0.77 | -0.88 | -1.37 | -0.34 | -0.31 | -1.06 | -0.62 | -0.21 | -0.76 | -1.02 | -1.38 | -0.81 |
| **F486** | 4.92 | 5.23 | 3.7 | 4.26 | 4.28 | 4.34 | 4.49 | 5.77 | 1.79 | 2.52 | 2.85 | 3.38 | 2.99 | -0.01 | 3.57 | 5.26 | 3.84 | 1.53 | 0.4 | 3.45 |
| **N487** | 0.03 | -0.18 | -0.02 | 0.1 | 0 | -0.11 | 0.09 | 0.04 | -0.18 | -0.12 | -0.04 | -0.14 | -0.1 | -0.36 | -0.22 | 0.03 | -0.01 | -0.55 | -0.26 | -0.18 |
| **C488** | -0.05 | -0.71 | -0.47 | 0.62 | -0.02 | -0.78 | -0.87 | -0.17 | -0.24 | -0.27 | 0.38 | -0.7 | -0.7 | 2.99 | -0.89 | -0.05 | -0.17 | -0.42 | -0.93 | -0.46 |
| **Y489** | 1.5 | 0.32 | 0.75 | 1.55 | 1.45 | 1.14 | 1.47 | 1.68 | 0.48 | 0.92 | 0.39 | 1.4 | 0.75 | -0.03 | 1.13 | 1.53 | 1.11 | -0.01 | -0.11 | 0.82 |
| **F490** | -0.15 | -0.1 | -0.01 | 0.15 | 0.27 | 0.31 | 0.31 | 0.53 | 0.57 | 0.66 | 0.76 | 0.81 | 1.05 | 1.27 | 1.29 | 1.37 | 1.51 | 1.58 | 1.62 | 3.5 |
| **L492** | -0.02 | -0.15 | -0.02 | 0.36 | -0.06 | -0.07 | 0.23 | 0 | 0.01 | 0.15 | 0 | -0.14 | -0.03 | 0.16 | 0.02 | -0.06 | 0.19 | 0.14 | -0.51 | 0.04 |
| **Q493** | 1.46 | 1.76 | 0.67 | 2.89 | 1.36 | 0.01 | 2.74 | 1.51 | 0.8 | 1.3 | 0.97 | 1.75 | 1.07 | 0.27 | 1.27 | 1.36 | 1.26 | 0.44 | 0.59 | 0.87 |
| **S494** | -0.23 | 1.87 | 0.18 | 2.19 | -0.07 | 0.41 | 2.26 | 0.54 | 0.23 | 0.73 | 1.76 | 0.8 | -0.23 | -0.48 | 1.25 | 0.01 | 0.43 | 0.06 | -1.22 | -1.28 |
| **Y495** | -0.1 | -0.51 | -0.24 | 0.61 | -0.13 | -0.24 | 0.39 | -0.04 | -0.39 | 0 | -0.36 | -0.55 | -0.19 | 0.07 | -0.25 | -0.08 | -0.11 | 0.4 | 0.01 | -0.07 |
| **Q498** | 0.12 | -0.76 | 0.08 | 0.26 | 0.06 | 0 | 0.38 | 0.16 | -0.43 | -0.12 | -0.16 | -0.37 | -0.22 | -1.25 | -0.03 | 0.1 | 0.01 | -0.35 | -1.31 | -0.05 |
| **Y505** | 0.54 | 0.08 | 0.35 | 0.85 | 0.46 | 0.66 | 1.08 | 0.63 | 0.33 | -0.14 | -0.19 | 0.49 | 0.63 | -0.11 | 0.48 | 0.51 | 0.4 | 0.17 | 0.01 | 0.27 |

**Table S5. The predicted binding stabilities of SARS-CoV-2 RBD targeting P2B-2F6 by DS.**

|  | **A** | **R** | **N** | **D** | **C** | **Q** | **E** | **G** | **H** | **I** | **L** | **K** | **M** | **F** | **P** | **S** | **T** | **W** | **Y** | **V** |
| --- | --- | --- | --- | --- | --- | --- | --- | --- | --- | --- | --- | --- | --- | --- | --- | --- | --- | --- | --- | --- |
| **R346** | -0.53 | 0.00 | -0.53 | -1.09 | -0.52 | -0.59 | -1.17 | -0.53 | -0.59 | -0.57 | -0.61 | 0.07 | -0.58 | -0.54 | -0.58 | -0.52 | -0.59 | -0.68 | -0.54 | -0.54 |
| **Y351** | 0.06 | 0.40 | 0.10 | -0.47 | 0.03 | -0.03 | -0.61 | 0.05 | 0.01 | 0.06 | 0.05 | 0.70 | -0.06 | 0.04 | 0.04 | 0.06 | 0.04 | -0.17 | -0.01 | 0.09 |
| **V445** | 0.19 | -1.49 | -0.31 | -0.50 | 0.16 | -0.96 | -1.56 | 0.43 | 0.14 | -1.01 | -1.00 | 0.90 | -0.89 | -0.05 | -0.71 | 0.18 | 0.11 | -0.75 | -0.03 | -0.03 |
| **G447** | -0.03 | 0.65 | -0.11 | -0.60 | -0.16 | 0.83 | -0.71 | 0.00 | -0.13 | 0.67 | -0.22 | 0.72 | -0.12 | -0.24 | -0.19 | -0.14 | 0.20 | -0.17 | -0.27 | 0.07 |
| **N448** | 0.26 | 0.43 | 0.00 | -0.17 | 0.21 | -0.27 | -0.43 | 0.38 | -0.47 | -0.49 | -0.61 | 0.33 | -0.35 | 0.16 | -0.17 | 0.25 | -0.13 | -0.54 | -0.58 | 0.04 |
| **Y449** | 1.76 | 3.43 | 1.20 | 1.04 | 1.30 | 0.98 | 2.25 | 2.85 | 0.36 | 2.66 | 0.37 | 3.93 | 1.69 | -0.14 | 2.22 | 2.45 | 2.79 | -0.03 | 0.00 | 1.12 |
| **N450** | 0.62 | 1.07 | 0.02 | 0.33 | 0.53 | 0.71 | 0.74 | 0.82 | 0.39 | 0.29 | -0.95 | 1.23 | 0.60 | 0.42 | 1.59 | 1.29 | 0.66 | 0.12 | 0.51 | -0.07 |
| **L452** | 1.17 | 1.12 | 0.63 | 0.30 | 0.94 | 0.52 | 0.89 | 1.31 | 0.19 | 0.13 | 0.00 | 2.24 | 0.29 | 0.01 | 0.93 | 1.20 | 0.73 | -1.04 | -0.92 | 0.73 |
| **I472** | 0.36 | 0.27 | 0.37 | -0.49 | 0.39 | 0.00 | -0.60 | 0.39 | -0.02 | 0.00 | -0.23 | 0.42 | -0.06 | 0.13 | 0.28 | 0.37 | 0.30 | 0.31 | 0.15 | 0.20 |
| **V483** | 0.89 | 1.77 | 0.99 | 0.47 | 0.75 | 1.53 | 0.18 | 1.27 | 0.93 | -0.41 | -0.28 | 1.38 | 0.19 | 0.09 | 0.32 | 1.64 | 0.65 | 0.09 | 0.20 | -0.02 |
| **E484** | 1.14 | 1.63 | 1.14 | 0.41 | 1.03 | 0.97 | -0.03 | 1.74 | 0.99 | 0.33 | 0.34 | 2.31 | 1.24 | 0.51 | 1.02 | 1.58 | 1.56 | -0.62 | 0.83 | 0.54 |
| **G485** | -0.16 | 1.18 | 0.16 | -0.65 | -0.13 | 0.12 | -0.56 | -0.01 | 0.39 | 0.41 | 0.36 | 0.81 | -0.05 | -0.35 | -0.02 | 0.17 | 0.44 | -0.50 | -0.47 | 0.28 |
| **F486** | 0.02 | 0.69 | 0.02 | -0.67 | 0.05 | 0.03 | -0.60 | 0.06 | 0.04 | -0.03 | 0.01 | 0.70 | 0.02 | 0.01 | -0.12 | 0.07 | 0.01 | -0.90 | 0.01 | -0.02 |
| **F490** | 2.01 | 2.26 | 1.44 | 0.92 | 1.76 | 2.04 | 1.04 | 2.42 | 0.54 | 0.85 | 0.96 | 2.35 | 1.78 | 0.08 | 1.54 | 2.15 | 1.82 | 0.24 | 0.36 | 0.80 |
| **S494** | -0.03 | 1.87 | -0.28 | -0.64 | -0.23 | 0.73 | 0.52 | 0.30 | -0.22 | -0.42 | 0.35 | 1.99 | -0.02 | 0.01 | 1.25 | 0.00 | 0.21 | 0.33 | -0.02 | -0.03 |

**Table S6. The predicted binding stabilities of SARS-CoV-2 RBD targeting ACE2 by DS.**

|  | **A** | **R** | **N** | **D** | **C** | **Q** | **E** | **G** | **H** | **I** | **L** | **K** | **M** | **F** | **P** | **S** | **T** | **W** | **Y** | **V** |
| --- | --- | --- | --- | --- | --- | --- | --- | --- | --- | --- | --- | --- | --- | --- | --- | --- | --- | --- | --- | --- |
| **K417** | 1.06 | 0.51 | 1.47 | 2 | 1.15 | 1.19 | 2.38 | 1.01 | 0.9 | 0.88 | 1.01 | -0.02 | 1.22 | 0.73 | 1.1 | 1.04 | 1.13 | 0.68 | 0.29 | 1.07 |
| **G446** | 0.04 | -0.81 | -0.1 | 0.72 | 0.06 | 0.12 | 0.54 | 0.04 | -0.1 | -0.33 | -0.06 | -0.61 | -0.24 | 0 | -0.31 | 0.04 | -0.32 | -0.35 | 0.04 | -0.25 |
| **Y449** | 0.66 | 0.06 | 0.48 | 1.45 | 0.59 | 0.69 | 1.56 | 0.62 | 0.68 | 0.69 | 0.74 | 0.27 | 0.73 | 0.1 | 0.7 | 0.68 | 0.77 | 0.49 | -0.01 | 0.74 |
| **Y453** | 0.29 | -0.24 | 0.28 | 0.72 | 0.28 | -0.03 | 0.72 | 0.26 | 0.33 | 0.12 | 0.21 | -0.34 | 0.04 | 0.06 | 0.32 | 0.34 | 0.56 | -0.26 | 0 | 0.16 |
| **L455** | 1.87 | 1.54 | 1.07 | 2.88 | 1.7 | 0.55 | 2.11 | 2 | 0.26 | 0.24 | -0.1 | 1.05 | 1.12 | 0.64 | 1.37 | 1.74 | 0.49 | 1.42 | -0.14 | 1.03 |
| **F456** | 2.12 | 1.18 | 1.53 | 2.52 | 1.83 | 1.49 | 2.45 | 2.21 | 0.51 | 1.36 | 0.85 | 0.13 | 0.46 | -0.16 | 1.85 | 1.97 | 2.2 | 0.71 | 0.76 | 1.88 |
| **A475** | 0 | 1.73 | -0.94 | 1.36 | -0.6 | -0.61 | 2 | 0.56 | -0.51 | -0.76 | 0.18 | 0.23 | 0.39 | 1.15 | -0.51 | -0.84 | -0.89 | -0.06 | 1.69 | -0.75 |
| **F486** | 2.01 | 0.77 | 2.01 | 2.73 | 2.04 | 1.6 | 2.69 | 2.04 | 0.81 | 1.17 | 0.82 | 1.34 | 1.72 | -0.26 | 1.59 | 2.34 | 1.84 | 0.39 | -0.11 | 1.35 |
| **N487** | 0.89 | -2.19 | 0.49 | 1.08 | 0.39 | -0.33 | 0.66 | 1.04 | 1.06 | -1.02 | -0.13 | -1.44 | -0.68 | 1.69 | 0.93 | 0.78 | 0.29 | 0.52 | 2.22 | -0.48 |
| **Y489** | 2.48 | -0.34 | 1.92 | 2.54 | 2.26 | 1.49 | 2.63 | 2.71 | 1.35 | 0.77 | 1.13 | 1.2 | 1.38 | 0.39 | 2.06 | 2.47 | 1.24 | 1.2 | -0.24 | 0.83 |
| **Q493** | -0.11 | -0.73 | -0.68 | 0.26 | -0.09 | -0.1 | 0.06 | 0.04 | -0.78 | -1.33 | -1.12 | -0.88 | -0.61 | -1.48 | -0.32 | -0.14 | 0.27 | -1.13 | -1.96 | -0.3 |
| **G496** | -0.08 | 0.68 | 0.19 | 1.73 | -0.41 | 0.01 | 1.59 | 0 | 0.22 | 0.66 | 1.51 | -0.64 | 1.22 | 1.13 | -0.81 | -0.42 | -0.53 | -0.14 | 0.74 | -0.17 |
| **Q498** | 0.99 | -2.85 | 0.67 | 1.79 | 0.64 | 0.03 | 2.63 | 1.42 | -0.53 | 0.17 | 0.62 | -2.39 | 0.82 | -0.77 | 0.92 | 1.05 | 1.1 | -0.26 | 0.02 | 0.08 |
| **T500** | -0.35 | -2.83 | -0.86 | 1.24 | 0.44 | 0.48 | 1.64 | 0.56 | 0.34 | -1.28 | -0.61 | -1.15 | -0.3 | -0.24 | -1.18 | 0.97 | 0 | -0.2 | -1.66 | -0.72 |
| **N501** | 0.89 | 0.37 | 0.1 | 2.11 | 0.74 | -0.52 | 1.18 | 1.36 | 0.77 | 0.01 | -0.05 | -0.1 | -0.02 | 4.62 | -0.36 | 0.85 | 1.13 | 7.51 | 1.48 | -0.25 |
| **G502** | 2.29 | 3.39 | 4.83 | 6.53 | 5.32 | 5.76 | 6.47 | 0.05 | 4.85 | 5.43 | 4.88 | 3.95 | 4.82 | 4.9 | 34.51 | 5.57 | 5.13 | 5.2 | 5.95 | 5.76 |
| **Y505** | 1.76 | 2.79 | 0.64 | 2.15 | 1.48 | 1.71 | 3.02 | 2.27 | 0.38 | 2.55 | 0.78 | 1.56 | 1.09 | -0.12 | 2.11 | 2.01 | 2.52 | -0.43 | 0.06 | 1.43 |

**Table S7. The predicted binding stabilities of SARS-CoV-2 RBD targeting H11-D4 by Mutabind2.**

|  | **A** | **R** | **N** | **D** | **C** | **Q** | **E** | **G** | **H** | **I** | **L** | **K** | **M** | **S** | **T** | **W** | **Y** | **V** | **F** | **P** |
| --- | --- | --- | --- | --- | --- | --- | --- | --- | --- | --- | --- | --- | --- | --- | --- | --- | --- | --- | --- | --- |
| **R346** | 0.03 | 0.14 | 0.11 | -0.07 | -0.01 | -0.03 | -0.02 | -0.01 | 0.2 | 0.16 | 0.22 | -0.01 | 0.06 | -0.17 | -0.2 | 0.04 | 0.1 | 0.03 | 0.35 | 0.82 |
| **K444** | -0.25 | -0.02 | -0.33 | 0.23 | 0.17 | 0.22 | 0.26 | 0.25 | 0.01 | 0.08 | 0.18 | 0.12 | 0 | -0.76 | -0.79 | 0.11 | -0.65 | -0.26 | -0.33 | 1.33 |
| **T470** | 0.38 | 0.47 | 0.29 | -0.06 | 0.3 | 0.45 | 0.08 | 0.35 | 0.28 | 0.35 | 0.11 | 0.2 | 0.09 | 0.24 | 0.22 | 0.25 | -0.06 | 0.2 | -0.02 | 0.56 |
| **G482** | 0.42 | 0.11 | 0.27 | 0.47 | -0.17 | 0.08 | 0.44 | 0.25 | 0.51 | 0.31 | 0.38 | -0.08 | 0.22 | 0.38 | 0.25 | 0.46 | 0.43 | 0.27 | 0.44 | -0.37 |
| **V483** | -0.12 | 0.01 | 0.09 | 0.14 | -0.42 | 0 | 0.21 | 0.11 | 0.12 | 0.13 | -0.05 | -0.03 | -0.1 | 0.12 | 0.06 | 0.16 | 0.33 | 0.16 | 0.2 | 0.31 |
| **E484** | 2.07 | 3.38 | 1.89 | 2.18 | 2.1 | 2.24 | 0.38 | 2.94 | 2.67 | 1.6 | 2.1 | 3.39 | 1.13 | 1.54 | 2.35 | 3.16 | 2.78 | 2.6 | 2.84 | 1.55 |
| **Y489** | 0.69 | 0.46 | 0.51 | 1.04 | 0.26 | 0.63 | 0.62 | 0.66 | 0.64 | 0.37 | 0.27 | 0.38 | 0.27 | 0.79 | 0.73 | 0.21 | 0.27 | 0.25 | 0.01 | 0.71 |
| **F490** | 1.35 | 1.66 | 1.25 | 1.62 | 0.92 | 1.49 | 1.59 | 1.13 | 0.58 | 1.65 | 1.25 | 1.48 | 0.78 | 1.46 | 1.22 | -0.13 | 0.07 | 1.75 | 0.36 | 2.07 |
| **P491** | 0.51 | 0.49 | 0.34 | 0.41 | 0.72 | 0.31 | 0.1 | 0.5 | 0.1 | 0.65 | 0.63 | 0.33 | 0.64 | 0.5 | 0.24 | 0.24 | 0.67 | 0.65 | 0.8 | -0.15 |
| **L492** | 1.01 | 1.63 | 0.98 | 0.85 | 0.9 | 0.9 | 0.94 | 1.23 | 0.91 | 1.01 | 0.04 | 1.46 | 0.04 | 1.04 | 1.16 | 0.81 | 0.73 | 1 | 0.97 | 0.77 |
| **Q493** | 0.54 | 1.25 | 0.96 | 0.73 | 0.63 | 0.57 | 0.55 | 1.68 | 1.46 | 1.54 | 0.74 | 0.88 | 0.61 | 1.12 | 0.97 | 2.08 | 1.66 | 1.02 | 1.77 | 1.93 |
| **S494** | -0.46 | 1.64 | 1.14 | 0.92 | -0.73 | 0.93 | 2.29 | 0.19 | 1.28 | 0.94 | 1.96 | 2.32 | 1.53 | -0.05 | 0.93 | 3.06 | 2.88 | 1.1 | 2.98 | 2.74 |
| **G446** | -0.06 | 0.82 | -0.19 | 0.52 | 0.35 | 0.52 | 0.45 | 0.25 | 0.73 | -0.09 | 0.44 | 1.44 | 0.28 | -0.14 | -0.29 | 0.71 | 0.66 | -0.2 | 0.47 | -0.58 |
| **Y449** | 1.72 | 1.68 | 0.83 | 1.88 | 1.63 | 1.45 | 1.85 | 1.76 | 0.76 | 2.45 | 0.99 | 1.68 | 0.82 | 1.73 | 1.65 | -0.55 | 0.33 | 1.76 | 0.42 | 0.96 |
| **N450** | 0.8 | 0.94 | 0.3 | 0.54 | 1.04 | 0.59 | 0.53 | 0.77 | 0.47 | 1.05 | 0.73 | 1.02 | 0.55 | 0.48 | 0.72 | 0.68 | 0.78 | 0.95 | 0.86 | 0.59 |
| **L452** | 0.87 | -0.28 | 0.2 | 0.95 | 0.63 | 0.16 | 0.19 | 0.8 | 0.34 | 0.64 | 0.13 | 0.08 | 0.1 | 0.53 | 0.53 | -1.11 | 0.13 | 0.51 | 0.44 | 0.13 |
| **L455** | 0.5 | 0.13 | 0.36 | 0.42 | 0.49 | 0.4 | 0.44 | 0.73 | 0.21 | -0.01 | 0.18 | 0.4 | 0.11 | 0.62 | 0.37 | -0.81 | -0.6 | 0.08 | -0.36 | 0.52 |
| **F456** | 0.45 | 0.32 | 0.2 | 0.5 | 0.33 | 0.54 | 0.56 | 0.54 | 0.42 | 0.08 | -0.17 | 0.54 | 0.17 | 0.4 | 0.2 | 0.26 | 0.2 | 0.27 | 0.27 | 0.57 |

**Table S8. The predicted binding stabilities of SARS-CoV-2 RBD targeting VH1-2-15 by Mutabind2.**

|  | **A** | **N** | **D** | **C** | **Q** | **E** | **G** | **H** | **I** | **L** | **K** | **M** | **F** | **P** | **S** | **T** | **W** | **Y** | **V** | **R** |
| --- | --- | --- | --- | --- | --- | --- | --- | --- | --- | --- | --- | --- | --- | --- | --- | --- | --- | --- | --- | --- |
| **G446** | -0.71 | 0.26 | 0.94 | -0.41 | 0.81 | 0.75 | 0.30 | 0.79 | 0.25 | 0.75 | 0.90 | 0.08 | 0.14 | 0.20 | 0.22 | -0.47 | 0.76 | 0.42 | -0.03 | 0.71 |
| **Y449** | 2.11 | 2.54 | 2.86 | 2.19 | 2.42 | 2.57 | 2.27 | 2.57 | 2.24 | 1.18 | 3.19 | 1.23 | 0.72 | 1.03 | 1.88 | 2.37 | 1.53 | 0.22 | 2.18 | 2.73 |
| **N450** | 0.22 | 0.20 | 0.19 | 0.19 | 0.08 | 0.14 | 0.23 | 0.17 | 0.14 | 0.18 | 0.21 | 0.18 | 0.17 | 0.09 | 0.26 | 0.14 | 0.09 | 0.07 | 0.25 | 0.87 |
| **L452** | -0.40 | -1.12 | -0.28 | -0.04 | -0.90 | -0.83 | -0.53 | 0.07 | -0.13 | 0.44 | -0.66 | -0.73 | 0.61 | -1.07 | -0.63 | -0.58 | 0.71 | 0.42 | -0.49 | -0.84 |
| **L455** | 0.48 | 0.40 | 0.56 | 0.58 | 0.75 | 0.58 | 1.28 | 0.10 | 0.40 | 0.19 | 0.66 | 0.03 | -0.95 | 0.66 | 0.78 | 0.90 | -0.22 | -1.11 | -0.21 | 0.97 |
| **F456** | 1.41 | 0.86 | 1.30 | 0.62 | 1.65 | 1.64 | 1.41 | 0.73 | 0.24 | -0.60 | 1.20 | -0.90 | 0.34 | 1.20 | 1.12 | 1.13 | 0.46 | 0.26 | 0.49 | 1.56 |
| **G482** | 0.23 | 0.13 | 0.33 | -0.05 | 0.27 | 0.18 | 0.16 | 0.09 | 0.07 | 0.02 | 0.11 | -0.10 | -0.44 | -0.66 | 0.18 | -0.36 | -0.45 | -0.40 | 0.09 | 0.21 |
| **V483** | -0.08 | -0.13 | 0.17 | -0.50 | 0.01 | 0.06 | 0.07 | 0.16 | 0.04 | -0.07 | 0.18 | 0.12 | 0.14 | -0.52 | 0.19 | 0.19 | 0.07 | -0.02 | 0.24 | -0.21 |
| **E484** | -2.32 | 2.09 | 1.88 | -1.35 | 0.58 | 0.91 | -0.59 | 1.43 | 2.32 | 0.41 | 0.41 | -0.34 | 2.64 | -3.41 | -1.38 | 0.53 | 2.55 | 2.31 | 1.45 |  |
| **G485** | 1.43 | 0.46 | 1.16 | -0.08 | 1.12 | 0.54 | 0.14 | 1.62 | 1.36 | 0.64 | 0.42 | 0.96 | 0.56 | 2.24 | 0.60 | 1.91 | 0.95 | 0.60 | 1.43 | 1.10 |
| **C488** | 1.01 | 1.04 | 2.06 | -0.09 | 0.94 | 2.20 | 1.08 | 0.96 | 0.90 | 0.87 | 1.01 | 1.10 | 0.98 | 0.88 | 0.98 | 0.97 | 1.05 | 1.01 | 1.04 | 1.20 |
| **Y489** | 0.71 | 0.46 | 0.54 | 0.26 | 0.70 | 0.87 | 0.79 | 0.82 | 0.23 | 0.43 | 0.81 | 0.31 | 0.58 | 0.52 | 0.69 | 0.23 | 0.88 | 0.58 | 0.20 | 0.63 |
| **F490** | 3.03 | 2.04 | 2.83 | 2.87 | 1.88 | 2.24 | -0.32 | 2.56 | 2.84 | 1.34 | 1.56 | 1.15 | 0.54 | 2.75 | 3.04 | 2.21 | -1.31 | 0.49 | 2.63 | 2.64 |
| **L492** | 0.61 | 0.85 | 1.08 | 0.86 | 0.72 | 0.83 | 0.89 | 0.85 | 0.02 | -0.09 | 1.20 | 0.09 | 0.69 | 1.90 | 0.79 | 0.59 | 0.78 | 0.82 | -0.03 | 1.12 |
| **Q493** | -0.14 | -0.22 | 1.52 | 0.89 | 1.18 | 0.43 | 0.89 | 0.54 | -0.19 | 0.61 | 1.27 | 0.60 | 1.21 | 1.65 | -0.29 | -0.60 | 1.49 | 1.15 | -1.36 | 0.59 |
| **S494** | -0.85 | 0.94 | -0.34 | -0.13 | 0.57 | 1.54 | -0.70 | 2.19 | 1.81 | 2.23 | 2.02 | 1.04 | 2.31 | 1.06 | -0.86 | 1.56 | 2.30 | 2.29 | 1.58 | 2.22 |
| **G496** | 1.89 | 1.53 | 2.24 | 1.01 | 1.36 | 2.54 | 0.03 | 2.29 | 1.48 | 2.47 | 1.87 | 1.03 | 1.24 | 2.29 | 1.03 | 1.12 | 1.25 | 0.98 | 1.25 | 2.04 |
| **Q498** | 0.44 | 0.52 | 0.42 | 0.42 | 0.31 | 0.16 | 0.31 | 0.60 | 0.42 | 0.38 | 0.29 | 0.36 | 0.63 | 0.58 | 0.16 | 0.28 | 0.63 | -0.03 | 0.54 | 0.34 |

**Table S9. The predicted binding stabilities of SARS-CoV-2 RBD targeting SR4 by Mutabind2.**

|  | **A** | **N** | **D** | **C** | **Q** | **E** | **G** | **H** | **I** | **L** | **K** | **M** | **F** | **P** | **S** | **T** | **W** | **Y** | **V** | **R** |
| --- | --- | --- | --- | --- | --- | --- | --- | --- | --- | --- | --- | --- | --- | --- | --- | --- | --- | --- | --- | --- |
| **V445** | 0.32 | -0.45 | 0.3 | 0.25 | 0.13 | 0.17 | 0.35 | -0.05 | 0.03 | -0.14 | -0.04 | -0.18 | -0.19 | -0.44 | 0 | -0.03 | -0.28 | -0.45 | 0.24 | -0.06 |
| **G446** | -0.21 | -0.71 | 0.25 | -0.05 | 0.76 | 0.15 | 0.23 | 0.83 | -0.69 | -0.12 | 1 | -0.89 | -0.16 | -0.89 | -0.1 | -0.45 | 0.7 | 0.04 | -0.62 | 1.29 |
| **G447** | 0.61 | 0.27 | 1.84 | 1.21 | 0.99 | 1.52 | 0 | 1.41 | 0.64 | 0.6 | 1.35 | 0.89 | 0.85 | 0.57 | 0.99 | 1.01 | 0.42 | 0.84 | 1.07 | 1.27 |
| **Y449** | 2.89 | 2.53 | 3.06 | 3.12 | 3.04 | 2.48 | 3.28 | 2.53 | 2.95 | 3.12 | 3.11 | 2.64 | 0.61 | 2.04 | 2.87 | 2.84 | 0.94 | 0.41 | 2.8 | 3.17 |
| **L452** | 0.7 | 0.15 | 0.99 | 0.74 | 0.25 | 0.78 | 0.86 | 0.42 | 0.8 | 0.16 | -0.48 | 0.22 | 0.54 | -0.26 | 0.57 | 0.62 | -0.95 | 0.8 | 0.72 | -0.33 |
| **Y453** | 0.58 | 0.46 | 0.62 | 0.65 | 0.63 | 0.56 | 0.72 | 0.73 | 0.65 | 0.69 | 0.84 | 0.68 | 0.22 | 0.72 | 0.68 | 0.65 | 0.64 | 0.29 | 0.65 | 0.87 |
| **L455** | 0.62 | 0.79 | 1.27 | 0.17 | 0.76 | 0.86 | 1.02 | 0.68 | 0.51 | 0.26 | 0.43 | -0.24 | -0.57 | 0.99 | 0.94 | 0.6 | -0.73 | -0.86 | 0.63 | 0.46 |
| **F456** | 0.69 | 0.61 | 0.96 | 0.87 | 0.91 | 0.77 | 0.9 | 0.81 | 0.36 | -0.06 | 0.88 | 0.25 | 0.2 | 0.85 | 0.59 | 0.57 | 0.32 | 0.14 | 0.47 | 0.88 |
| **E484** | 0.16 | 0.3 | 0.24 | 0.26 | 0.27 | 0.32 | 0.25 | 0.36 | 0.12 | -0.04 | 0.28 | -0.05 | 0.25 | -0.52 | -0.02 | 0.13 | 0.41 | 0.27 | 0.25 | 0.32 |
| **Y489** | 1.04 | 1.06 | 1.08 | 0.61 | 0.85 | 0.82 | 1.46 | 0.89 | 0.48 | 0.55 | 1.21 | 0.44 | 0.09 | 1.22 | 1.08 | 0.98 | 0.21 | 0.38 | 0.51 | 0.85 |
| **F490** | 0.41 | 0.46 | 0.73 | 0.12 | 0.54 | 0.86 | -0.09 | 0.44 | 0.57 | 0.4 | 0.49 | 0.23 | 0.35 | 0.47 | 0.32 | 0.26 | 0.47 | 0.22 | 0.61 | 0.43 |
| **L492** | 0.8 | 0.88 | 0.76 | 0.56 | 0.88 | 0.91 | 0.78 | 0.71 | 0.69 | 0.01 | 0.75 | -0.23 | 0.9 | 0.77 | 0.66 | 0.85 | 0.37 | 0.75 | 0.88 | 1.21 |
| **Q493** | 1.91 | 0.58 | 2.08 | 1.66 | 0.4 | 2.15 | 1.93 | 1.98 | 0.52 | 0.47 | 1.22 | 0.04 | 1.76 | 2.34 | 1.7 | 1.04 | 2.29 | 1.88 | 1.05 | 0.73 |
| **S494** | 0.23 | 1.66 | 1.66 | 0.1 | 2.18 | 2.28 | 1.34 | 2.56 | 2.01 | 0.96 | 1.21 | 1.16 | 2.34 | 0.77 | 0.17 | 0.69 | 2.76 | 2.4 | 1.76 | 1.81 |
| **Y495** | 0.47 | 0.78 | 1.07 | 0.59 | 0.51 | 1.08 | 0.73 | 0.51 | 0.08 | 0.59 | 0.93 | 0.78 | 0.11 | 0.65 | 0.76 | 0.69 | 0.55 | 0.11 | 0.39 | 0.82 |
| **G496** | 1.91 | 2.44 | 2.59 | 2.41 | 2.84 | 3.46 | 0.24 | 2.52 | 2.19 | 2.48 | 2.8 | 2.76 | 2.59 | 2.6 | 2.23 | 2.4 | 2.61 | 2.82 | 2.41 | 2.67 |
| **Q498** | 1.09 | -0.02 | 0.79 | -0.01 | 0.07 | 0.8 | 1.79 | 0.89 | -0.07 | 0.81 | -0.07 | -0.46 | 0.8 | 1.44 | 1.12 | 0.7 | 1.46 | 0.67 | 0.19 | 1.13 |
| **T500** | 0.15 | -0.03 | 0.09 | -0.04 | 0.16 | 0.13 | 0.04 | 0.16 | 0.12 | -0.01 | 0.15 | -0.05 | -0.05 | 0.23 | 0.01 | 0.24 | 0.34 | 0.42 | 0.19 | 0.33 |
| **N501** | 0.01 | 0.06 | 0.27 | 0.51 | 0.44 | 0.61 | 0.98 | 1.08 | 0.93 | 0.67 | 0.88 | 0.09 | -1.28 | 1.57 | -0.26 | -0.3 | 0.98 | -0.29 | 0.27 | 0.78 |
| **G502** | 0.65 | 0.36 | 0.75 | 0.37 | 0.31 | 0.81 | 0.22 | 0.17 | 0.23 | 0.09 | 1.04 | -0.1 | 0.08 | -0.29 | 0.14 | 0.63 | 0.22 | 0.32 | 0.62 | 1.03 |
| **Y505** | 0.43 | 0.82 | 0.98 | 0.37 | 0.4 | 0.87 | 0.55 | 0.2 | 0.85 | 0.29 | 0.66 | 0.31 | 0.14 | 1 | 0.95 | 0.33 | 0.05 | 0.3 | 0.88 | 1.02 |

**Table S10. The predicted binding stabilities of SARS-CoV-2 RBD targeting MR17 by Mutabind2.**

|  | **A** | **N** | **D** | **C** | **Q** | **E** | **G** | **H** | **I** | **L** | **K** | **M** | **F** | **P** | **S** | **T** | **W** | **Y** | **V** | **R** |
| --- | --- | --- | --- | --- | --- | --- | --- | --- | --- | --- | --- | --- | --- | --- | --- | --- | --- | --- | --- | --- |
| **R403** | 0.5 | -0.2 | 0.76 | 0.83 | 0.61 | 1.51 | 0.74 | 1.06 | 1.37 | 0.9 | -1.08 | 0.39 | 0.91 | -0.52 | -0.06 | 1.2 | 0.88 | 0.53 | 1.03 | 0 |
| **K417** | -0.11 | 0.25 | 0.92 | 0.25 | 0.12 | 0.27 | 0.32 | 0.35 | -0.23 | -0.24 | -0.01 | -0.2 | -0.43 | -0.22 | 0.04 | -0.05 | 0.14 | -0.41 | -0.35 | 0.02 |
| **G446** | -0.36 | -0.56 | 0.14 | -0.11 | 0.46 | 0.49 | 0.03 | 0.37 | -0.41 | -0.03 | 0.11 | -0.27 | 0.03 | -0.56 | -0.31 | -0.47 | 0.47 | 0.45 | -0.41 | 0.45 |
| **Y453** | 2.94 | 1.35 | 1.63 | 2.83 | 2.86 | 2.71 | 2.77 | 2.88 | 2.87 | 1.8 | 1.79 | 2.25 | 1.25 | 2.88 | 2.83 | 2.91 | 3 | 0.02 | 1.32 | 2.88 |
| **L455** | 2.17 | 0.23 | 1.53 | 0.89 | 1.06 | 1.41 | 2.38 | 0.82 | 0.31 | -0.07 | 1.13 | -0.63 | -0.45 | 2.24 | 2.2 | 1.86 | -1.4 | -0.96 | 2.02 | 1.49 |
| **F456** | 0.5 | 0.45 | 0.79 | 0.69 | 0.62 | 0.72 | 0.65 | 0.77 | 0.26 | 0.23 | 0.42 | 0.2 | 0.16 | 0.89 | 0.41 | 0.01 | 0.2 | 0.22 | -0.07 | 0.28 |
| **I472** | 0.2 | 0.22 | 0.94 | -0.22 | 0.23 | 0.68 | 0.14 | 0.47 | 0.02 | -0.02 | 0.91 | 0.1 | 0.04 | -0.02 | 0.13 | 0.1 | 0.13 | 0.13 | 0.2 | 1.07 |
| **E484** | 0.19 | 0.78 | 0.18 | 0.63 | 0.8 | -0.02 | 0.92 | 1.49 | 0.49 | 1.47 | 0.34 | 1 | 1.94 | 0.15 | 0.29 | 0.21 | 1.96 | 1.76 | 0.29 | 0.76 |
| **G485** | 0.33 | 0.13 | 0.48 | -0.35 | 0.42 | 0.47 | -0.2 | 0.47 | 1.27 | 0.43 | 0.38 | 0.64 | 0.44 | 0.03 | 0.18 | 0.53 | 1.08 | 0.91 | 0.82 | 0.51 |
| **F486** | 1.75 | 1.83 | 2.16 | 1.74 | 2.12 | 2.2 | 2.36 | 1.58 | 1.52 | 1.45 | 1.71 | 0.45 | -0.05 | 1.55 | 1.44 | 1.58 | 1.61 | 1.51 | 2.14 | 2.28 |
| **N487** | 0.5 | -0.06 | 0.62 | 0.45 | 0.45 | 0.4 | 0.43 | 0.21 | 0.29 | 0.27 | 0.25 | 0.25 | 0.31 | 0.21 | 0.51 | 0.47 | 0.3 | 0.19 | 0.43 | 0.33 |
| **C488** | 3.17 | 1.94 | 1.79 | 0.2 | 1.7 | 2.59 | 1.61 | 1.74 | 1.44 | 1.8 | 2.36 | 1.62 | 2.35 | 1.79 | 2.72 | 2.08 | 1.62 | 1.85 | 1.87 | 2.45 |
| **Y489** | 1.51 | 1.59 | 2.12 | 0.91 | 1.25 | 2.04 | 2.03 | 1.43 | 0.98 | 0.8 | 1.33 | 1.03 | 0.19 | 2.02 | 1.6 | 1.56 | 0.66 | 0.43 | 1.26 | 1.19 |
| **F490** | 1.06 | 1.21 | 1.93 | 0.44 | 1.27 | 1.83 | 1.01 | 1.24 | 0.71 | 0.51 | 1.96 | 0.4 | 0.21 | 1.84 | 1.11 | 0.72 | 0.18 | 0.5 | 1.01 | 1.64 |
| **L492** | 0.45 | 0.52 | 0.7 | -0.09 | 0.6 | 0.78 | 0.35 | 0.48 | -0.33 | -0.18 | 0.57 | -0.49 | 0.58 | 0.41 | 0.07 | 0.46 | 0.38 | 0.41 | -0.25 | 0.73 |
| **Q493** | 0.79 | 0.38 | 1.85 | 0.75 | 0.12 | 2.11 | 1.54 | 1.02 | 1.18 | 0.33 | 1.49 | 0.39 | 0.19 | 1.73 | 1.1 | 0.27 | 0.99 | 1 | 0.45 | 1.48 |
| **S494** | 0.25 | 0.85 | 1.31 | -1.26 | 0.59 | 2.04 | 1.1 | 0.12 | 1.13 | 0.42 | 0.46 | -0.17 | 0.72 | -1.01 | 0.17 | 0.34 | 0.91 | 0.53 | 0.86 | 0.78 |
| **Y495** | 1.07 | 1.14 | 1.46 | 1.28 | 0.98 | 1.49 | 1.63 | 0.98 | 1.05 | 1.09 | 1.26 | 1.49 | 0.13 | 1.77 | 1.76 | 1.01 | 1.63 | 0.01 | 1.03 | 0.89 |
| **Q498** | -0.11 | -0.1 | 0.11 | 0.06 | 0.06 | 0.23 | 0.2 | -0.17 | -0.26 | 0.05 | -0.55 | -0.37 | -0.15 | 0.31 | 0.15 | -0.32 | 0.42 | -1.06 | -0.42 | -0.05 |
| **Y505** | 0.19 | 0.1 | 0.45 | 0.17 | 0.31 | 0.31 | -0.19 | 0.19 | 0.47 | 0.19 | 0.27 | 0.13 | 0.16 | 0.2 | 0.27 | 0.08 | 0.23 | 0.05 | 0.71 | 0.25 |
|  |  |  |  |  |  |  |  |  |  |  |  |  |  |  |  |  |  |  |  |  |

**Table S11. The predicted binding stabilities of SARS-CoV-2 RBD targeting P2B-2F6 by Mutabind2.**

|  | **A** | **R** | **N** | **D** | **C** | **Q** | **E** | **G** | **H** | **I** | **L** | **K** | **M** | **F** | **P** | **S** | **T** | **W** | **Y** | **V** |
| --- | --- | --- | --- | --- | --- | --- | --- | --- | --- | --- | --- | --- | --- | --- | --- | --- | --- | --- | --- | --- |
| **R346** | 0.13 | 0.15 | 0.13 | 0.09 | 0.21 | -0.43 | 0.16 | 0.1 | 0.15 | 0.18 | 0.21 | -0.07 | 0.12 | 0.36 | 0.22 | -0.25 | 0.14 | 0.44 | 0.21 | 0.1 |
| **Y351** | 0.65 | 1.17 | 1.07 | 1.18 | 0.76 | 0.78 | 1 | 0.91 | 0.83 | 0.64 | 0.31 | 1.22 | 0.39 | 0.12 | 1.01 | 1.02 | 0.59 | 0.25 | 0.22 | 0.36 |
| **V445** | 0.22 | -0.08 | -0.77 | 0.22 | 0.21 | 0.11 | 0.11 | 0.25 | -0.21 | -0.11 | 0.11 | -0.3 | 0.01 | -0.57 | -0.11 | -0.26 | -0.28 | -0.44 | -0.84 | 0.22 |
| **G447** | 1.68 | 1.37 | 0.7 | 1.52 | 1.57 | 1.63 | 1.76 | 0.2 | 1.34 | 1.79 | 1.34 | 1.39 | 1.56 | 1.53 | 1.11 | 1.14 | 1.74 | 1.36 | 1.47 | 2.15 |
| **N448** | 0.73 | 0.65 | 0.03 | 0.57 | 0.65 | 0.59 | 0.66 | 0.69 | 0.4 | 0.62 | 0.74 | 0.89 | 0.61 | 0.69 | 0.74 | 0.38 | 0.53 | 1.6 | 0.45 | 0.62 |
| **Y449** | 1.44 | 2.43 | 1.47 | 2.41 | 1.64 | 1.6 | 2.25 | 2.01 | 1.83 | 1.35 | 0.76 | 1.24 | 0.47 | 0.23 | 1.71 | 1.77 | 2.11 | -0.37 | 0.03 | 2.4 |
| **N450** | 0.71 | 0.92 | 0.37 | 0.64 | 0.87 | 0.5 | 1.29 | 0.91 | 1.1 | 0.55 | 0.4 | 0.72 | 0.7 | 0.58 | 0.98 | 0.54 | 0.57 | 1.11 | 0.74 | 0.66 |
| **L452** | 0.91 | -0.81 | -0.05 | 1.28 | 0.75 | -0.08 | 1.01 | 0.88 | 0.41 | 0.9 | 0.05 | -0.14 | -0.18 | 0.05 | -0.31 | 0.68 | 0.69 | -1.36 | 0.51 | 0.45 |
| **I472** | 0.63 | 1.29 | 0.61 | 1.03 | -0.36 | 0.74 | 0.78 | 0.78 | 0.88 | 0.11 | 0.15 | 1.29 | 0.36 | 0.8 | 0.57 | 0.52 | 0.55 | 1.4 | 0.74 | 0.07 |
| **V483** | -0.26 | 0.24 | 0.13 | 0.13 | -0.77 | 0.12 | 0.13 | -0.01 | 0.17 | 0.15 | 0.08 | 0.1 | 0.11 | 0.04 | 0.24 | 0.15 | 0.11 | 0.11 | 0.12 | 0.15 |
| **E484** | 0.83 | 0.58 | 0.45 | 0.66 | 0.84 | 0.94 | 0.24 | 1.66 | 1.05 | 1.22 | 1.24 | 0.88 | 1.5 | 1.11 | 0.07 | 0.42 | 0.86 | 1.64 | 0.92 | 1.1 |
| **G485** | 0.48 | 0.36 | 0.39 | 0.28 | 0.34 | 0.44 | 0.48 | 0.21 | 0.34 | 0.44 | 0.42 | 0.34 | 0.41 | 0.34 | 0.68 | 0.44 | 0.49 | 0.65 | 0.37 | 0.36 |
| **F486** | -0.18 | 0.01 | -0.1 | -0.07 | -0.16 | -0.07 | -0.02 | -0.11 | -0.07 | -0.05 | -0.06 | 0 | -0.07 | -0.07 | -0.04 | -0.44 | -0.2 | -0.11 | -0.03 | -0.03 |
| **F490** | 1.22 | 1.81 | 1.14 | 2.62 | 0.43 | 1.34 | 1.52 | -0.23 | 1.01 | 1.46 | 1.41 | 1.52 | 0.47 | 0.38 | 2.14 | 1.26 | 0.09 | 0.7 | 0.04 | 1.59 |
| **S494** | -0.01 | 0.58 | 0.37 | -0.33 | -1 | 0.08 | 0.34 | 0.06 | 0.7 | 0.01 | 0.54 | 0.34 | -0.23 | 0.59 | -0.02 | 0.14 | 0.09 | 0.57 | 0.63 | 0.04 |

**Table S12. The predicted binding stabilities of SARS-CoV-2 RBD targeting ACE2 by Mutabind2.**

|  | **A** | **R** | **N** | **D** | **C** | **Q** | **E** | **G** | **H** | **I** | **L** | **K** | **M** | **F** | **P** | **S** | **T** | **W** | **Y** | **V** |
| --- | --- | --- | --- | --- | --- | --- | --- | --- | --- | --- | --- | --- | --- | --- | --- | --- | --- | --- | --- | --- |
| **K417** | -0.04 | 0.21 | 0.67 | 0.83 | 0.73 | 0.73 | 0.59 | 0.7 | 0.84 | -0.11 | -0.08 | 0.23 | -0.19 | -0.24 | 0.8 | 0.19 | 0.28 | 0.7 | -0.59 | -0.1 |
| **G446** | -0.16 | 0.56 | -0.34 | 0.39 | 0.19 | 0.59 | 0.65 | 0.18 | 0.53 | -0.43 | 0.14 | 0.3 | -0.08 | 0.39 | -0.26 | -0.23 | -0.62 | 0.71 | 0.55 | -0.58 |
| **Y449** | 0.84 | 0.41 | 0.47 | 0.34 | 0.83 | 0.44 | 0.86 | 0.95 | 0.85 | 0.86 | 0.43 | 0.55 | 0.53 | 0.41 | 0.46 | 0.48 | 0.85 | -0.57 | 0.24 | 0.98 |
| **Y453** | 0.93 | 1.21 | 0.68 | 1.31 | 0.98 | 1.03 | 1.44 | 0.78 | 0.46 | 1.15 | 0.69 | 0.69 | 0.59 | -0.16 | 0.77 | 1.01 | 0.94 | -0.1 | 0.1 | 0.86 |
| **L455** | 2.05 | 2.34 | 1.91 | 2.4 | 1.95 | 2.15 | 1.27 | 2.42 | 1.53 | 0.58 | 0.31 | 2.56 | 0.18 | 1.67 | 2.63 | 2.33 | 2.28 | 0.65 | 0.42 | 2.43 |
| **F456** | 2.18 | 1.55 | 1.92 | 2.24 | 2.27 | 1.38 | 2.14 | 2.23 | 1.52 | 1.05 | 1.27 | 1.79 | 0.2 | 0.11 | 2.32 | 2.13 | 1.37 | 1.23 | 1.81 | 1.24 |
| **A475** | 0.02 | 0.56 | 0.15 | 1.15 | -0.22 | 0.8 | 0.26 | 0.57 | 0.93 | 0.92 | 1.29 | 1.07 | 0.31 | 1.47 | -0.1 | 0.68 | 0.88 | 1.28 | 1.53 | 1.04 |
| **F486** | 1.27 | 1.18 | 1.23 | 1.97 | 1.15 | 1.55 | 1.72 | 1.78 | 1.38 | 0.65 | 0.3 | 0.74 | 0.06 | 0.04 | 1.03 | 1.09 | 1.01 | 0.02 | -0.12 | 1.33 |
| **N487** | 1.4 | 1 | 0.04 | 1.19 | 0.67 | 1.46 | 1.76 | 1.01 | 1.27 | 1.47 | 0.61 | 0.68 | 0.76 | 1.33 | 1.84 | 1.41 | 1.28 | 1.81 | 1.34 | 1.44 |
| **Y489** | 2.22 | 1.77 | 1.95 | 1.86 | 0.97 | 1.77 | 1.75 | 1.97 | 2.58 | 1.36 | 0.91 | 1.59 | 1.29 | 0.26 | 1.92 | 1.97 | 0.69 | 0.38 | 0.52 | 1.42 |
| **Q493** | 0.55 | 0.16 | 0.07 | 0.79 | 0.63 | 0.88 | 0.89 | 0.98 | 0.05 | 0.45 | 0 | 0.11 | 0 | -0.02 | 1.1 | -0.11 | 0.3 | 0.39 | -0.05 | 0.42 |
| **G496** | 0.74 | 2.46 | 1.38 | 1.04 | 0.73 | 0.65 | 1.54 | 0 | 1.95 | 1.63 | 1.65 | 0.63 | 0.85 | 1.41 | 1.04 | 1.28 | 2.02 | 1.17 | 2.21 | 1.67 |
| **Q498** | 0.65 | -0.02 | 0.28 | 1.37 | -0.46 | 0.26 | 0.39 | 1.28 | 0.34 | -0.63 | -0.84 | -1.17 | -1.02 | -1.67 | 0.87 | 0.65 | 0.32 | 0.57 | -1.83 | 0.59 |
| **T500** | 0.41 | 0.51 | 0.21 | 0.57 | 0.42 | 0.28 | 0.81 | 0.59 | 0.46 | 0.68 | 0.61 | 0.35 | 0.18 | 0.57 | 0.4 | 0.37 | 0.34 | 1.14 | 0.84 | 0.38 |
| **N501** | -0.45 | 0.67 | 0.05 | 0.93 | -0.16 | 0.86 | 0.5 | 0.68 | 0.79 | 0.81 | -0.42 | 0.85 | -0.03 | 0.64 | 0.77 | -0.4 | -0.59 | 1.5 | 0.62 | -0.17 |
| **G502** | 1.39 | 0.88 | 1.11 | 1.54 | 1.29 | 0.55 | 1.36 | 0.03 | 0.88 | 0.78 | 1.01 | 0.97 | 1.01 | 1.08 | 2.16 | 1.26 | 1.2 | 1.11 | 1 | 1.02 |
| **Y505** | 1.15 | 1.14 | 1.51 | 1.98 | 0.78 | 1.68 | 1.59 | 1.07 | 0.56 | 1.06 | 1.06 | 1.28 | 0.95 | 0.43 | 1.46 | 1.52 | 1.2 | 0.44 | 0.16 | 1.64 |

**Table S13. The predicted binding stabilities of SARS-CoV-2 RBD targeting H11-D4 by FoldX.**

|  | **A** | **R** | **N** | **C** | **D** | **Q** | **E** | **G** | **H** | **I** | **L** | **K** | **M** | **F** | **P** | **S** | **T** | **W** | **Y** | **V** |
| --- | --- | --- | --- | --- | --- | --- | --- | --- | --- | --- | --- | --- | --- | --- | --- | --- | --- | --- | --- | --- |
| **R346** | 0.25 | 0 | 0.46 | 0.02 | -0.28 | -0.28 | 0.04 | 0.42 | 0.1 | -0.2 | -0.07 | -0.21 | -0.54 | -0.61 | 1.73 | -0.04 | -0.48 | -0.95 | -0.23 | 0.41 |
| **K444** | 0.39 | -0.34 | 0.84 | 0.28 | 0.38 | -0.12 | 0.45 | 0.75 | 0.3 | 0.33 | 0.04 | 0 | -0.06 | -0.1 | 3.34 | -0.49 | 0.27 | 0.38 | 0.09 | 4.72 |
| **G446** | 3.1 | 3.71 | 2.69 | 3.33 | 3.9 | 3.41 | 3.86 | 0 | 3.3 | 4.95 | 4.31 | 3.93 | 3.87 | 3.96 | 6.69 | 3.42 | 4.6 | 4.2 | 3.7 | 1.42 |
| **Y449** | 1.65 | 1.36 | 1.14 | 1.81 | 3.21 | 1.93 | 3.15 | 1.49 | 1.28 | 3.45 | 0.69 | -0.1 | 0.82 | 0.15 | 1.19 | 2.65 | 2.44 | -0.05 | 0 | -0.14 |
| **N450** | -0.3 | -0.21 | 0 | -0.11 | -0.2 | -0.54 | -0.6 | 0.47 | 1.26 | -0.13 | -1.51 | -0.23 | -1.56 | 0.1 | 1.23 | -0.2 | -0.27 | 0.06 | -0.88 | 1.54 |
| **L452** | 2.79 | 1.69 | 2.62 | 2.25 | 2.52 | 2.16 | 1.5 | 3.87 | 3.3 | 2.23 | 0 | 1.46 | 0.38 | 1.17 | 3.8 | 3.17 | 2.03 | 1.87 | 1.75 | 0.8 |
| **L455** | 1.87 | 1.64 | 1.32 | 1.68 | 1.71 | 1.36 | 1.83 | 2.79 | 0.93 | 0.55 | 0 | 1.22 | 1.4 | 0.6 | 5.38 | 2.13 | 1.41 | 1.13 | 0.95 | 2.36 |
| **F456** | 3.18 | 1.88 | 3.51 | 2.88 | 3.78 | 1.6 | 2.59 | 4.17 | 1.4 | 3.12 | 0.3 | 1.59 | 0.36 | 0 | 4.9 | 3.92 | 3.24 | -0.15 | 0.37 | -0.14 |
| **T470** | 0.16 | 0.04 | 0.29 | -0.27 | -0.68 | -0.31 | -0.41 | 0.71 | -0.1 | -0.28 | -1.15 | -0.21 | -1.2 | -1.13 | 1.66 | 0.93 | 0 | -1.44 | -2.05 | 4.07 |
| **G482** | 2.53 | 2.14 | 1.72 | 2.17 | 1.75 | 2.03 | 1.92 | 0 | 1.9 | 3.8 | 1.81 | 1.97 | 1.81 | 1.78 | 4.54 | 2.29 | 2.69 | 1.63 | 1.81 | 0 |
| **V483** | 0.19 | -0.62 | -0.33 | -0.01 | -0.14 | -0.4 | -0.1 | 0.23 | 0 | -0.17 | -0.05 | -0.61 | -0.21 | -0.07 | 3.48 | 0 | -0.37 | 0.05 | -0.03 | 0 |
| **E484** | 4.64 | 9.13 | 4.35 | 4.47 | 3.85 | 2.94 | 0 | 5.9 | 6.3 | 2.8 | 1.59 | 7.77 | 2.22 | 6.58 | 4.97 | 5.06 | 5.27 | 10.11 | 9.83 | 3.65 |
| **Y489** | 2.62 | 1.39 | 2.28 | 2.34 | 4.14 | 2.14 | 2.5 | 3.89 | 1.88 | 1.18 | -0.39 | 1.47 | 0.1 | -0.42 | 3.46 | 3.65 | 2.85 | 0.08 | 0 | 0.79 |
| **F490** | 3.24 | 5.21 | 2.96 | 3.13 | 1.63 | 3.07 | 2.05 | 4.91 | 1.49 | 4.27 | 0.84 | 3.59 | 0.64 | 0 | 5.33 | 4.48 | 4.81 | 0.19 | 0.23 | 4.79 |
| **P491** | 2.63 | 3.56 | 3.44 | 1.72 | 4.49 | 3.9 | 4.09 | 3.42 | 25 | 3.54 | 3.92 | 3.58 | 2.12 | 19.59 | 0 | 2.73 | 3.54 | 14.58 | 19.87 | 2.81 |
| **L492** | 3.73 | 4.45 | 2.88 | 3.03 | 3.47 | 2.68 | 2.72 | 4.88 | 3.5 | 2.32 | 0 | 3.71 | 0.96 | 2.75 | 5.2 | 4.78 | 4.47 | 6.49 | 4.38 | 2.85 |
| **Q493** | 1.21 | 1.09 | 1.3 | 0.82 | 0.09 | 0 | -1.48 | 2.01 | 5.1 | 2.18 | -0.2 | 0.71 | -0.01 | 5.58 | 5.72 | 0.15 | -0.29 | 8.38 | 6.18 | 1.23 |
| **S494** | -1.95 | 6.36 | 5.98 | -0.48 | 5.92 | 7.31 | 6.45 | -0.3 | 67 | 4.76 | 8.99 | 6.59 | 4.05 | 16.18 | 4.93 | 0 | 2.28 | 18.65 | 16.32 | 2.01 |

**Table S14. The predicted binding stabilities of SARS-CoV-2 RBD targeting VH1-2-15 by FoldX.**

|  | **A** | **R** | **N** | **C** | **D** | **Q** | **E** | **G** | **H** | **I** | **L** | **K** | **M** | **F** | **P** | **S** | **T** | **W** | **Y** | **V** |
| --- | --- | --- | --- | --- | --- | --- | --- | --- | --- | --- | --- | --- | --- | --- | --- | --- | --- | --- | --- | --- |
| **G446** | 3.18 | 3.56 | 2.97 | 3.85 | 2.83 | 2.53 | 2.83 | 0.00 | 2.90 | 4.87 | 2.69 | 3.01 | 3.51 | 1.59 | 6.29 | 3.84 | 3.66 | 2.60 | 1.77 | 3.66 |
| **Y449** | 1.69 | 3.49 | 2.06 | 1.63 | 3.05 | 2.33 | 2.52 | 1.74 | 2.26 | 2.27 | 0.66 | 1.99 | 0.72 | 0.83 | 0.95 | 1.64 | 2.44 | 6.55 | 0.00 | 2.44 |
| **N450** | -0.44 | -0.05 | 0.00 | -0.23 | -0.22 | 0.12 | -0.37 | -0.10 | 0.32 | -0.21 | -0.58 | -0.28 | -0.61 | -0.26 | -0.61 | -0.07 | 0.34 | -0.19 | -0.11 | 0.34 |
| **L452** | 2.27 | 2.39 | 2.02 | 2.06 | 1.87 | 2.59 | 2.24 | 3.17 | 1.90 | 3.21 | 0.54 | 2.24 | 0.93 | 1.26 | 7.86 | 2.45 | 2.46 | 2.10 | 1.73 | 2.46 |
| **L455** | 1.05 | 3.03 | -0.73 | 0.98 | -0.04 | 0.87 | 0.05 | 2.16 | 0.49 | 3.12 | 0.00 | 1.60 | 0.39 | -0.29 | 3.37 | 1.60 | 1.44 | 1.79 | 0.27 | 1.44 |
| **F456** | 2.52 | 2.73 | 2.18 | 2.21 | 2.55 | 2.39 | 2.35 | 3.75 | 1.58 | 5.31 | 0.15 | 1.85 | 0.92 | 0.00 | 5.76 | 3.49 | 5.35 | 1.09 | 0.61 | 5.35 |
| **G482** | 3.57 | 2.77 | 3.63 | 3.63 | 4.12 | 3.14 | 3.91 | 0.00 | 3.70 | 4.71 | 3.46 | 3.18 | 3.08 | 3.22 | 4.81 | 3.68 | 5.01 | 3.88 | 3.01 | 5.01 |
| **V483** | 0.05 | -0.42 | -0.66 | -0.05 | -0.42 | -0.35 | -0.48 | -0.56 | 0.19 | -0.38 | -0.20 | -0.54 | -0.11 | -0.29 | 0.79 | -0.35 | -0.07 | -0.29 | -0.31 | 2.88 |
| **E484** | -1.76 | 1.26 | 2.41 | -0.37 | 3.17 | 0.16 | 0.00 | -3.01 | 4.30 | 3.00 | -1.67 | -0.36 | -3.12 | 2.82 | -4.70 | -0.38 | 2.88 | 8.35 | 2.80 | 8.91 |
| **G485** | 3.93 | 4.80 | 4.10 | 4.12 | 2.69 | 4.53 | 4.41 | 0.00 | 4.70 | 7.17 | 4.53 | 4.74 | 4.73 | 2.53 | 10.54 | 4.38 | 8.91 | 5.41 | 3.42 | 3.63 |
| **C488** | 1.12 | 4.18 | 2.65 | 0.00 | 5.66 | 6.29 | 7.72 | 1.19 | 32.10 | 6.23 | 7.07 | 3.76 | 3.13 | 13.40 | 6.90 | 1.06 | 3.63 | 17.59 | 18.96 | -1.38 |
| **Y489** | 0.49 | 0.08 | -1.67 | -0.54 | -1.15 | -0.66 | -1.37 | -0.37 | 0.39 | -0.26 | -0.76 | 0.02 | -1.22 | -0.27 | -1.16 | -0.28 | -1.38 | 0.11 | 0.00 | 1.22 |
| **F490** | 3.39 | 1.47 | 2.77 | 3.01 | 4.28 | 2.78 | 3.84 | 3.68 | 2.23 | 2.58 | 1.39 | 1.11 | 1.11 | 0.00 | 4.37 | 2.05 | 1.22 | 0.87 | 0.27 | 3.54 |
| **L492** | 3.14 | 2.81 | 3.47 | 3.26 | 3.55 | 1.46 | 1.96 | 3.74 | 2.04 | 1.53 | 0.00 | 2.57 | 0.76 | 0.99 | 2.97 | 3.31 | 3.54 | 4.92 | 2.82 | -0.38 |
| **Q493** | -0.27 | -1.54 | -0.33 | -0.27 | -0.08 | 0.00 | 0.12 | 0.29 | 0.65 | -0.38 | -1.01 | -0.05 | -1.18 | -0.54 | 3.46 | -0.24 | -0.38 | 2.20 | -0.42 | 3.42 |
| **S494** | 0.94 | 4.76 | 5.16 | 0.97 | 5.19 | 5.83 | 6.60 | 1.77 | 18.00 | 5.40 | 6.62 | 3.18 | 1.12 | 8.63 | 1.72 | 0.00 | 3.42 | 16.33 | 9.11 | 5.20 |
| **G496** | 3.75 | 5.01 | 5.64 | 4.06 | 4.58 | 4.24 | 3.98 | 1.18 | 15.00 | 8.07 | 6.09 | 5.43 | 4.77 | 8.76 | 11.13 | 1.03 | 5.20 | 9.87 | 9.09 | 7.25 |
| **Q498** | 0.13 | 0.21 | 0.51 | 0.01 | 0.32 | 0.00 | -0.47 | 0.01 | 2.04 | 1.13 | 0.09 | 0.31 | -0.19 | 2.28 | 2.82 | -0.33 | 1.38 | 3.49 | 2.03 | 0.60 |

**Table S15. The predicted binding stabilities of SARS-CoV-2 RBD targeting SR4 by FoldX.**

|  | **A** | **R** | **N** | **C** | **D** | **Q** | **E** | **G** | **H** | **I** | **L** | **K** | **M** | **F** | **P** | **S** | **T** | **W** | **Y** | **V** |
| --- | --- | --- | --- | --- | --- | --- | --- | --- | --- | --- | --- | --- | --- | --- | --- | --- | --- | --- | --- | --- |
| **V445** | 0.23 | -0.10 | 0.15 | 0.37 | 0.46 | -0.04 | 0.92 | 0.51 | 0.40 | -0.22 | -0.82 | -0.28 | -1.03 | -0 | -1.29 | 0.39 | 0.12 | -0.20 | 0.08 | 0.00 |
| **G446** | 2.50 | 2.06 | 1.80 | 2.26 | 1.15 | 2.13 | 8.68 | 0.00 | 9.10 | 4.14 | 2.15 | 2.31 | 1.39 | 6.7 | 5.09 | 2.37 | 4.50 | 7.91 | 6.53 | 4.53 |
| **G447** | 5.88 | 12.00 | 8.95 | 7.51 | 10.33 | 7.66 | 3.38 | 0.00 | 47.00 | 10.55 | 6.80 | 10.65 | 6.48 | 17 | 13.81 | 5.57 | 5.96 | 23.78 | 20.78 | 9.54 |
| **Y449** | 4.77 | 5.21 | 2.72 | 4.14 | 4.00 | 4.12 | 1.82 | 5.26 | 4.15 | 3.63 | 3.67 | 3.60 | 2.36 | 0.2 | 4.04 | 4.52 | 4.53 | 4.47 | 0.00 | 4.31 |
| **L452** | 2.70 | 1.00 | 2.06 | 2.34 | 3.90 | 1.24 | 4.63 | 3.74 | 1.72 | 2.16 | 0.00 | 0.45 | 0.77 | 1.2 | 4.85 | 2.95 | 3.13 | 0.63 | 1.25 | 2.27 |
| **Y453** | 3.78 | 3.31 | 3.66 | 3.57 | 5.80 | 3.26 | 0.83 | 5.02 | 2.30 | 2.70 | 1.30 | 2.67 | 1.52 | 0.6 | 5.67 | 3.95 | 3.67 | 0.70 | 0.00 | 2.57 |
| **L455** | 2.34 | 2.06 | 2.12 | 1.46 | 2.69 | 1.69 | 3.74 | 2.97 | 0.80 | 1.89 | 0.00 | 0.25 | 1.23 | 1.2 | 5.18 | 3.09 | 3.24 | 3.52 | 0.51 | 2.55 |
| **F456** | 3.97 | 3.74 | 3.83 | 3.34 | 4.80 | 2.69 | 0.00 | 4.71 | 1.99 | 2.69 | 1.22 | 2.83 | 0.90 | 0 | 4.92 | 4.48 | 3.85 | 0.31 | 0.78 | 2.16 |
| **E484** | 0.78 | 0.24 | 1.20 | 0.43 | 1.08 | 0.14 | 4.21 | 1.40 | 1.41 | -0.33 | -0.85 | -0.11 | -0.83 | 1.1 | -0.60 | 0.62 | 0.36 | 0.91 | 1.18 | 0.30 |
| **F489** | 4.19 | 3.94 | 4.23 | 3.84 | 5.78 | 4.40 | 2.57 | 5.75 | 3.33 | 1.72 | 2.39 | 4.27 | 3.01 | 0.3 | 6.95 | 4.09 | 3.05 | 2.31 | 0.00 | 2.63 |
| **F490** | 2.59 | 1.86 | 2.30 | 2.83 | 2.43 | 2.07 | 2.67 | 3.46 | 1.18 | 2.35 | 1.20 | 2.04 | 0.78 | 0 | 0.37 | 3.46 | 3.28 | 1.62 | 0.57 | 2.67 |
| **L492** | 3.54 | 3.85 | 2.98 | 3.01 | 3.88 | 3.09 | 0.15 | 4.92 | 3.37 | 2.30 | 0.00 | 2.67 | 0.86 | 2.6 | 6.47 | 4.83 | 4.29 | 5.58 | 3.54 | 2.87 |
| **Q493** | 0.84 | -0.32 | -0.40 | 0.46 | 0.30 | 0.00 | 7.27 | 1.80 | 3.90 | -0.74 | -1.65 | -1.00 | -1.59 | 2.4 | 4.12 | 1.07 | 0.06 | 9.30 | 5.95 | -0.58 |
| **S494** | 0.30 | 22.55 | 4.49 | 2.25 | 5.03 | 20.76 | 5.51 | 1.68 | 63.20 | 8.30 | 12.29 | 14.98 | 5.69 | 13 | 0.08 | 0.00 | 0.87 | 23.53 | 15.05 | 3.59 |
| **Y495** | 4.44 | 4.64 | 5.46 | 4.57 | 6.68 | 4.32 | 11.22 | 5.72 | 8.90 | 7.05 | 3.45 | 3.26 | 1.74 | 0.8 | 4.76 | 5.25 | 4.01 | 3.48 | 0.00 | 5.50 |
| **G496** | 4.97 | 11.97 | 8.74 | 8.56 | 11.99 | 8.39 | 0.39 | 0.00 | 8.90 | 11.93 | 10.20 | 10.62 | 6.45 | 7.9 | 15.40 | 8.72 | 10.73 | 16.71 | 11.30 | 9.73 |
| **Q498** | 0.79 | 5.02 | 0.06 | 0.27 | -0.12 | 0.00 | -0.65 | 2.19 | 2.20 | 0.73 | -1.04 | 3.39 | -0.78 | 3.8 | 2.92 | 0.75 | -0.68 | 11.92 | 4.64 | 0.57 |
| **T500** | -0.56 | -1.02 | -0.88 | -0.55 | -0.83 | -0.70 | -0.69 | -0.37 | 0.00 | -0.89 | -1.02 | -1.27 | -1.21 | -1 | -0.03 | -0.90 | 0.00 | -0.24 | -1.35 | 0.22 |
| **N501** | 0.27 | -0.37 | 0.00 | 0.30 | -0.68 | 0.87 | -0.78 | 1.35 | 0.00 | 1.17 | -1.14 | -0.20 | -1.30 | -2 | 5.89 | 0.55 | -0.05 | -0.46 | -2.06 | 0.59 |
| **G502** | -0.19 | -0.13 | -0.11 | 0.14 | -1.05 | -0.38 | 1.05 | 0.00 | 0.00 | -0.37 | -1.31 | -0.32 | -1.58 | -1 | -2.34 | -0.38 | 0.45 | -0.08 | -0.65 | 0.43 |
| **Y505** | 1.18 | 1.40 | 1.47 | 1.15 | 0.85 | 1.02 | 1.05 | 1.48 | 1.00 | 1.16 | -0.61 | 1.21 | 0.10 | -1 | 2.43 | 1.45 | 1.55 | 0.36 | 0.00 | 1.50 |

**Table S16. The predicted binding stabilities of SARS-CoV-2 RBD targeting MR17 by FoldX.**

|  | **A** | **R** | **N** | **C** | **D** | **Q** | **E** | **G** | **H** | **I** | **L** | **K** | **M** | **F** | **P** | **S** | **T** | **W** | **Y** | **V** |
| --- | --- | --- | --- | --- | --- | --- | --- | --- | --- | --- | --- | --- | --- | --- | --- | --- | --- | --- | --- | --- |
| **R403** | 1.34 | 0.00 | 3.17 | 1.66 | 4.75 | 1.53 | 3.64 | 1.94 | 7.10 | 3.54 | 1.10 | 0.83 | -0.06 | 7.87 | 4.19 | 1.91 | 5.16 | 6.70 | 3.80 | 9.49 |
| **K417** | 1.09 | -0.59 | 0.96 | 1.10 | 2.67 | 0.28 | 1.37 | 2.06 | 1.40 | 0.35 | 0.27 | 0.00 | 0.52 | 0.57 | -0.59 | 3.19 | 1.26 | 1.22 | 0.44 | 0.86 |
| **G446** | 2.84 | 2.59 | 2.98 | 2.98 | 2.98 | 2.65 | 2.89 | 0.00 | 2.80 | 3.54 | 2.76 | 2.62 | 2.15 | 1.96 | 5.67 | 5.78 | 3.83 | 2.25 | 3.83 | 2.21 |
| **Y453** | 5.37 | 1.63 | 5.93 | 4.81 | 7.94 | 4.76 | 7.03 | 6.40 | 3.80 | 4.78 | 2.49 | 3.46 | 2.72 | 0.71 | 6.68 | 2.54 | 5.26 | 4.82 | 6.05 | 0.00 |
| **L455** | 2.58 | 1.79 | 2.46 | 1.34 | 4.18 | 2.74 | 2.67 | 3.39 | 3.20 | 2.48 | 0.00 | 2.10 | 0.83 | 1.69 | 6.13 | 1.78 | 4.06 | 1.13 | 2.28 | 1.87 |
| **F456** | 1.35 | 1.66 | 2.87 | 2.63 | 2.29 | 1.84 | 2.29 | 2.79 | 2.60 | 3.90 | 1.23 | 2.55 | 0.84 | 0.00 | 6.96 | 0.64 | 1.20 | 0.80 | 0.96 | -0.04 |
| **I472** | 0.15 | 0.65 | 0.64 | 0.18 | 0.19 | 0.08 | -0.73 | 0.45 | 0.27 | 0.00 | 1.46 | 0.51 | -0.12 | -0.20 | -0.74 | 0.59 | 0.06 | 0.66 | -0.05 | -0.08 |
| **E484** | 0.80 | 1.22 | 1.73 | 0.73 | 1.00 | -0.15 | 1.20 | 1.48 | 3.50 | 0.64 | -0.18 | 0.79 | -0.08 | 2.82 | 1.90 | 0.86 | -0.20 | 2.93 | 0.16 | 1.58 |
| **G485** | 0.90 | 1.31 | -0.20 | 1.16 | -0.20 | 1.12 | 0.17 | 0.00 | 1.33 | 2.41 | 1.03 | 0.89 | 0.54 | 0.76 | -1.38 | 3.53 | 3.31 | 1.99 | 2.58 | 0.03 |
| **F486** | 2.59 | 2.95 | 3.37 | 2.99 | 2.82 | 2.89 | 2.86 | 3.36 | 3.10 | 1.40 | 0.59 | 2.48 | 0.33 | 0.00 | 1.51 | 1.50 | 2.92 | 2.15 | 2.18 | 0.82 |
| **N487** | 1.75 | 0.89 | 0.00 | 1.69 | 1.30 | 1.30 | 0.77 | 1.29 | 1.10 | 0.39 | 0.99 | 1.12 | 0.13 | 0.63 | 0.57 | 3.49 | 2.69 | 0.84 | 1.37 | 0.77 |
| **C488** | 1.90 | 11.08 | 4.13 | 0.00 | 3.40 | 7.43 | 6.74 | 3.00 | 24.00 | 1.57 | 3.26 | 7.79 | 6.52 | 12.79 | 6.87 | 3.23 | 3.11 | 25.42 | 2.48 | 18.15 |
| **Y489** | 2.09 | 2.55 | 2.97 | 1.97 | 2.68 | 1.35 | 2.29 | 3.64 | 2.90 | -0.11 | -0.11 | 2.46 | 1.10 | -0.51 | 5.13 | 2.47 | 2.32 | -0.42 | 0.61 | 0.00 |
| **F490** | 2.09 | 2.80 | 2.66 | 2.26 | 2.36 | 2.91 | 2.23 | 2.68 | 2.00 | 0.60 | 0.69 | 2.43 | 0.41 | 0.00 | 3.65 | 4.58 | 1.99 | 0.17 | 1.14 | 0.72 |
| **L492** | 3.15 | 3.79 | 2.73 | 2.81 | 3.41 | 2.09 | 2.57 | 4.48 | 3.50 | 1.15 | 0.00 | 2.20 | 0.55 | 3.46 | 4.98 | 1.47 | 3.22 | 6.44 | 1.65 | 5.42 |
| **Q493** | 0.99 | 1.30 | 1.57 | 0.55 | 3.68 | 0.00 | 2.66 | 2.61 | 1.20 | -0.29 | -1.2 | 0.46 | -1.04 | -0.83 | 6.06 | 0.00 | 0.63 | -0.98 | 0.14 | 0.81 |
| **S494** | 0.05 | -0.33 | 1.65 | 0.02 | 2.72 | 1.91 | 2.33 | 1.48 | 0.20 | 3.23 | -0.22 | 0.16 | -0.87 | -1.19 | -1.90 | 0.00 | 0.23 | 0.02 | 1.40 | -0.03 |
| **Y495** | 5.04 | 5.01 | 5.88 | 5.00 | 6.70 | 5.58 | 6.32 | 6.46 | 5.00 | 4.20 | 2.58 | 4.20 | 2.04 | 1.49 | 5.33 | 5.78 | 5.18 | 3.26 | 4.79 | 0.00 |
| **Q498** | -0.13 | -1.78 | 0.38 | -0.10 | 0.27 | 0.00 | 0.16 | -0.07 | 0.00 | -1.32 | -1.36 | -1.40 | -1.22 | -1.40 | 1.28 | 0.20 | -0.37 | -1.54 | -0.53 | -1.96 |
| **Y505** | 0.46 | 0.64 | -0.05 | 1.04 | 1.71 | 0.44 | 1.19 | 0.43 | 0.93 | 1.68 | 0.25 | 0.50 | 0.39 | 1.01 | -0.45 | 0.83 | 1.81 | 1.18 | 1.88 | 0.00 |

**Table S17. The predicted binding stabilities of SARS-CoV-2 RBD targeting P2B-2F6 by FoldX.**

|  | **A** | **R** | **N** | **C** | **D** | **Q** | **E** | **G** | **H** | **I** | **L** | **K** | **M** | **F** | **P** | **S** | **T** | **W** | **Y** | **V** |
| --- | --- | --- | --- | --- | --- | --- | --- | --- | --- | --- | --- | --- | --- | --- | --- | --- | --- | --- | --- | --- |
| **R346** | 0.45 | 0.00 | 0.41 | 0.36 | 0.29 | -0.13 | 0.11 | 0.55 | 0.56 | 0.14 | 0.29 | 0.26 | 0.21 | 0.21 | 0.89 | 0.42 | 0.12 | -0.06 | 0.00 | 0.24 |
| **Y351** | 0.51 | 1.86 | 3.03 | 1.42 | 2.01 | 0.87 | 1.06 | 2.28 | 2.50 | 0.74 | 0.46 | 1.31 | -0.70 | -0.41 | 3.32 | 1.81 | 1.15 | 0.06 | 0.00 | -0.39 |
| **V445** | -0.13 | -0.38 | 0.12 | 0.39 | -0.17 | -0.23 | -0.40 | -0.07 | 0.01 | -0.27 | -0.34 | -0.4 | -0.49 | -0.22 | -0.92 | 0.24 | 0.01 | -0.52 | -0.22 | 0.00 |
| **G447** | 2.94 | 2.82 | 5.32 | 3.09 | 5.81 | 3.37 | 2.86 | 0.00 | 4.50 | 6.67 | 4.55 | 2.51 | 2.93 | 7.43 | 4.47 | 2.77 | 6.72 | 3.72 | 3.83 | 5.68 |
| **N448** | 2.31 | 3.18 | 0.00 | 1.99 | 0.73 | 1.49 | 1.77 | 3.52 | 2.60 | 1.37 | 0.56 | 1.68 | 0.56 | 1.71 | 7.23 | 2.77 | 1.75 | 5.32 | 2.15 | 2.00 |
| **Y449** | 1.67 | 2.30 | 1.49 | 1.97 | 2.40 | 2.12 | 2.53 | 2.25 | 1.50 | 1.93 | 0.94 | 1.86 | 0.84 | 0.20 | 3.11 | 1.94 | 1.74 | 1.01 | 0.00 | 3.40 |
| **N450** | 1.07 | 1.54 | 0.00 | 1.72 | 0.69 | 0.47 | 1.13 | 1.95 | 0.18 | 0.23 | 0.22 | 1.79 | -0.14 | 1.07 | 2.35 | 1.29 | 1.00 | 1.50 | 0.13 | 1.21 |
| **L452** | 3.55 | 3.40 | 2.68 | 2.76 | 3.24 | 1.97 | 2.98 | 4.77 | 2.12 | 2.10 | 0.00 | 2.25 | 0.50 | 0.98 | 5.33 | 3.66 | 4.16 | 1.97 | 1.82 | 3.13 |
| **I472** | 2.62 | 3.81 | 3.44 | 2.21 | 3.95 | 2.80 | 3.01 | 3.55 | 3.20 | 0.00 | 1.47 | 2.4 | 1.28 | 0.80 | 2.64 | 3.82 | 2.31 | 2.60 | 2.70 | 0.83 |
| **V483** | 0.31 | 0.22 | 0.23 | 0.16 | -0.10 | 0.19 | 0.05 | 0.39 | 0.33 | -0.03 | 0.18 | 0.05 | 0.29 | 0.03 | 5.46 | 0.28 | -0.09 | 0.24 | 0.08 | 0.00 |
| **E484** | 1.52 | 1.22 | 0.98 | 1.42 | 1.07 | 1.37 | 0.00 | 2.36 | 1.53 | 0.38 | -0.01 | 1.49 | 0.31 | 1.15 | -0.24 | 1.89 | 0.85 | 1.78 | 1.19 | 0.96 |
| **G485** | 2.06 | 2.17 | 1.53 | 2.12 | 0.59 | 2.52 | 1.96 | 0.00 | 1.27 | 3.92 | 1.69 | 2.01 | 1.49 | 1.64 | 9.36 | 1.43 | 3.14 | 2.00 | 1.75 | 4.25 |
| **F486** | 0.23 | -0.22 | 0.06 | 0.05 | -0.15 | -0.23 | -0.27 | 0.30 | 0.20 | 0.05 | -0.05 | -0.5 | 0.13 | 0.00 | 0.17 | 0.25 | 0.01 | 0.14 | 0.05 | 0.06 |
| **F490** | 4.45 | 2.23 | 4.02 | 4.05 | 3.74 | 3.57 | 3.69 | 5.74 | 2.40 | 2.37 | 2.00 | 3.17 | 1.45 | 0.00 | 2.87 | 5.62 | 4.52 | 2.61 | 1.59 | 3.43 |
| **S494** | -0.27 | 2.60 | 1.24 | -0.39 | 1.77 | 1.64 | 2.06 | 1.28 | 12.00 | 0.62 | 1.28 | 1.56 | -0.19 | 3.16 | -0.08 | 0.00 | -0.05 | 1.37 | 1.82 | 1.30 |

**Table S18. The predicted binding stabilities of SARS-CoV-2 RBD targeting ACE2 by FoldX.**

|  | **A** | **R** | **N** | **D** | **C** | **Q** | **E** | **G** | **H** | **I** | **L** | **K** | **M** | **F** | **P** | **S** | **T** | **W** | **Y** | **V** |
| --- | --- | --- | --- | --- | --- | --- | --- | --- | --- | --- | --- | --- | --- | --- | --- | --- | --- | --- | --- | --- |
| **K741** | 0.85 | -0.98 | -0.56 | 1.51 | 0.74 | 0.54 | 0.33 | 1.89 | 1.52 | -0.16 | -0.17 | 0.00 | -0.01 | 0.06 | -1.00 | 1.36 | 0.85 | 1.10 | -1.06 | 0.11 |
| **G446** | 2.31 | 3.32 | 3.62 | 3.61 | 3.59 | 3.11 | 3.63 | 0.00 | 3.70 | 4.50 | 3.77 | 3.56 | 3.53 | 3.49 | 6.27 | 3.63 | 4.35 | 3.54 | 3.52 | 4.50 |
| **Y449** | 2.21 | 1.52 | 1.92 | 2.13 | 2.26 | 1.98 | 2.33 | 2.34 | 2.27 | 2.23 | 1.82 | 1.47 | 1.83 | 1.63 | 1.84 | 1.82 | 2.55 | 2.53 | 0.00 | 2.57 |
| **Y453** | 2.80 | 1.70 | 2.32 | 4.55 | 2.34 | 2.18 | 3.51 | 3.89 | 0.95 | 2.49 | 0.55 | 1.64 | -0.14 | -1.51 | 4.49 | 3.59 | 2.79 | -1.31 | 0.00 | 3.52 |
| **L455** | 3.33 | 5.05 | -0.05 | 1.67 | 2.32 | 4.67 | 2.00 | 4.35 | 3.20 | 2.22 | 0.00 | 3.28 | 1.41 | 6.60 | 6.19 | 3.31 | 3.91 | 7.39 | 9.90 | 3.62 |
| **F456** | 4.74 | 2.85 | 4.55 | 5.58 | 4.00 | 3.81 | 4.14 | 5.40 | 3.80 | 3.07 | 1.45 | 3.11 | 0.89 | 0.00 | 5.88 | 4.35 | 4.35 | 3.65 | 3.23 | 2.37 |
| **A475** | 0.00 | 4.01 | -0.32 | -1.05 | 0.49 | 1.41 | 0.89 | 1.48 | 21.00 | 6.73 | 3.69 | 3.41 | 1.31 | 23.38 | 5.51 | 1.37 | 2.37 | 22.72 | 17.38 | 2.75 |
| **F486** | 2.93 | 1.86 | 3.10 | 2.97 | 2.92 | 2.52 | 2.51 | 3.26 | 2.40 | 1.75 | 1.08 | 1.77 | 0.68 | 0.00 | 1.85 | 3.19 | 2.70 | 1.70 | -0.13 | 2.34 |
| **N487** | 2.06 | 1.75 | 0.00 | 0.45 | 0.88 | 2.30 | 2.02 | 1.00 | 42.30 | 2.74 | 0.15 | 1.32 | 0.27 | 6.95 | 6.84 | 2.03 | 3.34 | 4.68 | 8.75 | 2.56 |
| **Y489** | 3.26 | 2.76 | 3.41 | 4.38 | 3.11 | 3.08 | 3.61 | 4.49 | 2.50 | 0.59 | 0.31 | 3.97 | -0.03 | -0.80 | 4.35 | 2.49 | 0.97 | -0.44 | 0.00 | 1.83 |
| **Q493** | 0.21 | 0.38 | 0.22 | -0.41 | 0.38 | 0.00 | -0.05 | 1.06 | -0.03 | 0.30 | -1.30 | -0.46 | -0.87 | -0.68 | 2.63 | 0.86 | 0.45 | -0.08 | -1.56 | 0.58 |
| **G496** | -1.17 | 0.78 | 1.27 | 3.96 | 0.15 | 1.64 | 1.64 | 0.00 | 1.25 | -0.07 | -0.18 | 1.57 | -0.32 | 0.05 | -1.59 | -2.31 | 1.37 | -0.03 | -0.14 | 1.26 |
| **Q498** | -0.26 | 2.17 | 0.51 | 1.02 | -0.51 | 0.00 | 0.36 | 0.77 | -0.20 | -1.92 | -2.55 | -0.40 | -1.75 | -0.01 | -0.31 | -0.39 | -0.67 | 0.91 | 0.51 | -1.23 |
| **T500** | 0.02 | -0.85 | -0.65 | 0.88 | 0.24 | -0.76 | 0.23 | 0.79 | -0.90 | 0.21 | -1.47 | -1.23 | -1.83 | -1.74 | -0.44 | -0.10 | 0.00 | -1.63 | -1.30 | 0.29 |
| **N501** | -0.86 | 5.12 | 0.00 | -1.07 | -0.53 | 0.25 | -0.46 | 0.28 | 6.20 | 1.83 | -2.91 | 1.85 | -0.88 | 7.38 | 3.07 | -1.34 | -0.38 | 14.08 | 13.81 | 1.60 |
| **G502** | 1.90 | 2.27 | 2.65 | 3.21 | 2.69 | 3.55 | 3.93 | 0.00 | 3.60 | 3.80 | 2.38 | 2.48 | 2.23 | 2.80 | 12.59 | 2.91 | 4.36 | 3.82 | 3.04 | 4.26 |
| **Y505** | 1.77 | 2.27 | 2.30 | 3.12 | 1.86 | 2.75 | 3.02 | 2.54 | 2.70 | 1.82 | 0.86 | 2.48 | 1.15 | 0.55 | 3.35 | 2.62 | 2.73 | 0.81 | 0.00 | 2.13 |

**Table S19. The predicted binding stabilities of SARS-CoV-2 RBD targeting H11-D4 by mCSM-PPI2.**

|  | **A** | **R** | **N** | **D** | **C** | **Q** | **E** | **G** | **H** | **I** | **L** | **K** | **M** | **F** | **P** | **S** | **T** | **W** |
| --- | --- | --- | --- | --- | --- | --- | --- | --- | --- | --- | --- | --- | --- | --- | --- | --- | --- | --- |
| **R346** | -0.31 | NA | -0.29 | -0.45 | -0.36 | -0.16 | -0.45 | -0.45 | -0.06 | -0.22 | -0.24 | 0.26 | -0.29 | -0.07 | -0.98 | -0.24 | -0.28 | -0.02 |
| **K444** | -0.19 | -0.03 | -0.13 | -0.08 | -0.17 | -0.11 | -0.13 | -0.26 | -0.04 | -0.15 | -0.13 | NA | -0.13 | -0.07 | -0.42 | -0.09 | 0.26 | 0.14 |
| **G446** | -0.06 | -0.18 | 0.05 | 0.24 | -0.04 | 0.10 | 0.29 | NA | 0.00 | 0.05 | -0.02 | -0.07 | 0.05 | 0.09 | -0.02 | -0.03 | 0.00 | 0.42 |
| **Y449** | -1.76 | -1.63 | -1.69 | -2.06 | -1.71 | -1.86 | -1.98 | -1.92 | -1.35 | -1.72 | -1.65 | -1.77 | -1.79 | -0.46 | -3.32 | -1.84 | -1.79 | -0.59 |
| **N450** | -0.42 | -0.42 | NA | 0.68 | -0.15 | -0.39 | 0.01 | -0.28 | 0.04 | -0.34 | -0.33 | -0.86 | -0.42 | -0.24 | -0.07 | -0.24 | -0.25 | -0.18 |
| **L452** | -0.86 | -0.22 | -0.37 | -0.59 | -0.63 | -0.37 | -0.74 | -0.90 | -0.34 | -0.74 | NA | 0.38 | -0.94 | 0.89 | -0.93 | -0.43 | -0.34 | 1.13 |
| **L455** | -0.81 | -0.35 | -0.21 | -0.53 | -0.52 | -0.32 | -0.59 | -0.69 | -0.36 | -0.70 | NA | -0.28 | -0.87 | 0.57 | -1.06 | 0.55 | -0.22 | 0.13 |
| **F456** | -0.64 | -0.65 | -0.78 | -0.82 | -0.75 | -0.80 | -0.83 | -0.83 | 0.52 | -1.02 | -0.26 | -0.68 | -0.90 | NA | -1.76 | -0.69 | -0.66 | -0.24 |
| **Y470** | -0.18 | -0.11 | -0.10 | -0.04 | -0.25 | -0.15 | -0.03 | -0.25 | 0.01 | -0.10 | -0.11 | -0.15 | -0.15 | -0.08 | -0.21 | 0.10 | NA | 0.27 |
| **G482** | -0.12 | -0.15 | 0.03 | 0.31 | -0.16 | 0.07 | 0.33 | NA | -0.02 | -0.08 | -0.11 | 0.00 | -0.08 | -0.02 | -0.29 | -0.07 | -0.04 | 0.26 |
| **V483** | -0.17 | -0.41 | -0.09 | -0.15 | -0.08 | -0.21 | -0.12 | -0.20 | 0.03 | 0.12 | -0.08 | 0.00 | -0.14 | 0.18 | -0.88 | -0.15 | -0.17 | 0.47 |
| **E484** | -1.58 | -1.81 | -1.36 | 0.62 | -1.61 | -1.43 | NA | -1.49 | -0.72 | -1.27 | -1.23 | -1.74 | -1.36 | -0.46 | -1.33 | -1.46 | -1.51 | -0.26 |
| **Y489** | -0.75 | -0.59 | -0.67 | -0.78 | -0.66 | -0.67 | -0.76 | -0.76 | -0.84 | -0.83 | -0.79 | -0.61 | -0.86 | -0.39 | -0.85 | -0.64 | -0.64 | -0.08 |
| **F490** | -2.57 | -2.41 | -2.30 | -2.81 | -2.39 | -2.44 | -2.71 | -2.72 | -1.72 | -2.16 | -2.18 | -2.46 | -2.37 | NA | -3.92 | -2.37 | -2.44 | 1.68 |
| **P491** | -1.29 | -0.72 | -0.73 | -1.03 | -1.14 | -0.81 | -0.92 | -1.38 | -0.64 | -1.14 | -1.12 | -0.63 | -1.27 | -0.21 | NA | -0.64 | 1.07 | 0.06 |
| **L492** | -1.53 | -1.54 | -1.56 | -1.95 | -1.40 | -1.42 | -1.77 | -1.67 | -1.20 | -1.43 | NA | -1.44 | -1.34 | 0.88 | -2.27 | -1.52 | -1.54 | -0.31 |
| **Q493** | -1.30 | -1.37 | 1.05 | -0.93 | -1.20 | NA | -0.87 | -1.30 | -0.44 | -1.21 | -1.31 | -1.46 | -1.17 | -1.25 | -1.37 | 1.06 | -1.21 | -0.67 |
| **S494** | -0.38 | 0.02 | -0.07 | 1.43 | -0.18 | -0.22 | -0.29 | -0.69 | 0.32 | -0.35 | -0.36 | 0.07 | -0.33 | -0.01 | -0.54 | NA | 1.95 | 0.24 |

**Table S20. The predicted binding stabilities of SARS-CoV-2 RBD targeting VH1-2-15 by mCSM-PPI2.**

|  | **A** | **R** | **N** | **D** | **C** | **Q** | **E** | **G** | **H** | **I** | **L** | **K** | **M** | **F** | **P** | **S** | **T** | **W** | **Y** | **V** |
| --- | --- | --- | --- | --- | --- | --- | --- | --- | --- | --- | --- | --- | --- | --- | --- | --- | --- | --- | --- | --- |
| **G446** | -0.35 | -0.65 | -0.24 | -0.12 | -0.37 | -0.24 | 0.00 | NA | -0.34 | -0.39 | -0.46 | -0.43 | -0.36 | -0.04 | -0.56 | -0.21 | 0.12 | 0.10 | 0.00 | -0.34 |
| **Y449** | -1.53 | -1.58 | -1.48 | -2.11 | -1.58 | -1.68 | -1.96 | -1.77 | -1.09 | -1.38 | -1.38 | -1.68 | -1.49 | -0.62 | -2.95 | -1.62 | -1.58 | -0.40 | NA | -1.55 |
| **N450** | -0.20 | 0.16 | NA | 0.02 | -0.07 | -0.02 | 0.13 | -0.23 | 0.08 | -0.38 | -0.39 | -0.09 | -0.26 | -0.09 | -0.20 | 0.03 | -0.02 | 0.07 | -0.02 | -0.20 |
| **L452** | -0.65 | 0.40 | -0.03 | -0.41 | -0.46 | -0.01 | -0.30 | -0.61 | -0.20 | -0.40 | NA | 0.66 | -0.58 | 0.77 | -0.58 | -0.21 | -0.18 | 0.99 | 0.63 | -0.59 |
| **L455** | -0.44 | -0.21 | -0.14 | -0.45 | -0.41 | -0.35 | -0.56 | -0.58 | -0.33 | -0.56 | NA | -0.14 | -0.48 | 0.43 | -0.61 | 0.48 | -0.13 | -0.30 | 0.79 | -0.54 |
| **F456** | -0.57 | -0.33 | -0.44 | -0.58 | -0.62 | -0.41 | -0.61 | -0.64 | 0.17 | -0.80 | -0.46 | -0.40 | -0.64 | NA | -1.18 | -0.43 | -0.40 | -0.20 | 0.46 | -0.63 |
| **G482** | -0.42 | -0.51 | -0.20 | 0.01 | -0.46 | -0.26 | -0.02 | NA | -0.29 | -0.43 | -0.46 | -0.36 | -0.44 | -0.17 | -0.62 | -0.29 | -0.34 | 0.04 | -0.11 | -0.42 |
| **V483** | -0.37 | -0.50 | -0.34 | -0.30 | -0.23 | -0.16 | -0.20 | -0.55 | -0.12 | -0.04 | -0.37 | -0.35 | -0.30 | 0.04 | 0.94 | -0.15 | -0.12 | 0.51 | 0.24 | NA |
| **E484** | -1.17 | -0.68 | -0.85 | -0.19 | -0.91 | -0.63 | NA | -0.91 | -0.10 | -0.46 | -0.50 | -0.86 | -0.68 | -0.56 | 2.25 | -0.84 | -0.88 | -0.09 | -0.03 | -0.68 |
| **G485** | -0.23 | -1.35 | -1.02 | -1.27 | -1.13 | -1.12 | -1.09 | NA | -0.72 | -1.08 | -1.12 | -1.16 | -1.13 | -0.33 | -1.07 | -0.75 | -1.04 | -0.29 | -0.29 | -1.13 |
| **C488** | -1.13 | -1.07 | -1.31 | -0.90 | NA | -1.15 | -0.90 | -1.17 | -0.57 | -0.79 | -0.71 | -1.04 | -0.94 | -0.36 | -0.96 | -1.06 | -1.16 | -0.33 | -0.13 | -0.88 |
| **Y489** | -0.68 | -0.15 | -0.40 | -0.54 | -0.56 | -0.63 | -0.60 | -0.65 | -0.54 | -0.60 | -0.59 | -0.39 | -0.63 | -0.19 | -0.59 | -0.48 | -0.48 | 0.02 | NA | -0.61 |
| **F490** | -1.55 | -1.51 | -1.32 | -1.85 | -1.49 | -1.49 | -1.64 | -1.75 | -1.41 | -1.47 | -1.55 | -1.55 | -1.56 | NA | -2.12 | -1.35 | -1.36 | 1.25 | 0.90 | -1.54 |
| **L492** | -0.95 | -0.92 | -0.91 | -1.31 | -1.09 | -0.87 | -1.34 | -1.09 | -0.60 | -0.86 | NA | -0.86 | -0.71 | 0.65 | -1.47 | -0.85 | -0.86 | -0.26 | -0.18 | -0.99 |
| **Q493** | -0.98 | -0.87 | 0.75 | -0.51 | -0.88 | NA | -0.36 | -0.84 | -0.20 | -0.76 | -0.80 | -1.14 | -0.80 | -0.83 | -0.79 | 0.85 | -0.72 | -0.47 | -0.62 | -0.82 |
| **S494** | -0.64 | -0.35 | -0.39 | 1.52 | -0.61 | -0.54 | -0.49 | -0.84 | -0.25 | -0.73 | -0.89 | -0.30 | -0.66 | -0.77 | -0.42 | NA | 0.94 | -0.64 | -0.45 | -0.62 |
| **G496** | -0.65 | -1.26 | -0.87 | 0.69 | -1.19 | -1.07 | -0.60 | NA | -0.74 | -1.48 | -1.33 | -1.12 | -1.24 | -0.91 | -1.42 | -0.71 | -1.04 | -0.82 | -0.78 | -1.21 |

**Table S21. The predicted binding stabilities of SARS-CoV-2 RBD targeting SR4 by mCSM-PPI2.**

|  | **A** | **R** | **N** | **D** | **C** | **Q** | **E** | **G** | **H** | **I** | **L** | **K** | **M** | **F** | **P** | **S** | **T** | **W** | **Y** | **V** |
| --- | --- | --- | --- | --- | --- | --- | --- | --- | --- | --- | --- | --- | --- | --- | --- | --- | --- | --- | --- | --- |
| **V455** | -0.21 | 0.15 | 0.10 | -0.06 | -0.04 | 0.14 | -0.05 | -0.29 | -0.05 | -0.33 | -0.26 | 0.03 | -0.22 | 0.31 | -0.17 | -0.06 | 0.09 | 0.77 | 0.46 | NA |
| **G446** | -0.86 | -1.28 | -0.92 | -1.20 | -0.86 | -0.98 | -1.06 | NA | -0.67 | -0.71 | -0.69 | -1.13 | -0.78 | -0.20 | -0.73 | -0.79 | 0.33 | 0.32 | -0.22 | -0.77 |
| **GLY** | -0.66 | -1.17 | -0.72 | -0.89 | -1.03 | -0.99 | -0.99 | NA | -0.63 | -0.94 | -0.95 | -0.97 | -0.80 | -0.60 | -1.58 | -0.73 | -0.76 | -0.72 | -0.54 | -0.73 |
| **Y449** | -1.65 | -1.69 | -1.65 | -2.23 | -1.69 | -1.87 | -2.19 | -2.04 | -1.67 | -1.61 | -1.56 | -1.79 | -1.77 | -0.92 | -2.67 | -1.70 | -1.67 | -0.69 | NA | -1.73 |
| **L452** | -0.81 | -0.46 | -0.60 | -1.09 | -0.76 | -0.64 | -1.16 | -0.85 | -0.15 | -0.60 | NA | 0.36 | -0.92 | 1.05 | -0.94 | -0.49 | -0.54 | 1.08 | 0.91 | -0.78 |
| **Y453** | -0.69 | -0.74 | -0.61 | -0.95 | -0.66 | -0.84 | -0.91 | -0.82 | -0.66 | -0.68 | -0.70 | -0.76 | -0.69 | -0.42 | -1.77 | -0.78 | -0.71 | 0.00 | NA | -0.71 |
| **L455** | -0.80 | -0.55 | -0.39 | -0.98 | -0.68 | -0.58 | -0.98 | -0.82 | -0.44 | -0.67 | NA | -0.52 | -0.76 | 0.53 | -1.10 | 0.51 | -0.27 | 0.02 | 1.33 | -0.86 |
| **F456** | -1.02 | -0.95 | -1.00 | -1.15 | -1.08 | -0.96 | -1.20 | -1.19 | 0.44 | -0.98 | -0.65 | -0.96 | -0.95 | NA | -1.90 | -1.01 | -0.97 | -0.17 | 0.53 | -1.01 |
| **E484** | -0.32 | -0.12 | -0.34 | 0.09 | -0.39 | -0.27 | NA | -0.24 | 0.10 | -0.26 | -0.14 | -0.16 | -0.26 | 0.05 | 0.67 | -0.25 | -0.20 | 0.01 | 0.08 | -0.19 |
| **Y489** | -1.30 | -1.07 | -1.17 | -1.30 | -1.27 | -1.21 | -1.31 | -1.38 | -1.26 | -1.32 | -1.28 | -1.18 | -1.35 | -0.66 | -1.29 | -1.16 | -1.16 | -0.46 | NA | -1.28 |
| **F490** | -0.82 | -0.69 | -0.65 | -1.23 | -0.77 | -1.01 | -0.96 | -1.31 | -0.80 | -0.94 | -0.87 | -0.84 | -0.78 | NA | -1.78 | -0.72 | -0.68 | 0.99 | 0.61 | -0.81 |
| **L492** | -1.24 | -1.05 | -1.14 | -1.87 | -1.45 | -1.09 | -1.90 | -1.43 | -0.49 | -0.97 | NA | -1.02 | -1.05 | 1.10 | -1.69 | -1.10 | -1.11 | 0.16 | 0.33 | -1.07 |
| **Q493** | -1.51 | -0.95 | NA | -1.09 | -1.35 | NA | -0.77 | -1.74 | -0.20 | -0.99 | -0.98 | -1.13 | -0.87 | -0.80 | -1.15 | 1.27 | -1.16 | -0.52 | -0.55 | -1.08 |
| **S494** | -0.92 | -0.43 | 1.27 | 1.48 | -0.74 | -0.61 | -0.31 | -1.17 | -0.08 | -0.68 | -0.90 | -0.50 | -0.77 | -0.64 | -0.81 | NA | 1.37 | -0.43 | -0.45 | -0.71 |
| **Y495** | -0.56 | -0.59 | -0.55 | -0.73 | -0.50 | -0.67 | -0.69 | -0.61 | -0.75 | -0.61 | -0.63 | -0.60 | -0.63 | -0.26 | -1.22 | -0.61 | -0.58 | -0.21 | NA | -0.60 |
| **G496** | -1.04 | -1.45 | -0.58 | 1.25 | -1.34 | -1.25 | -0.95 | NA | -0.51 | -1.29 | -1.25 | -1.34 | -1.20 | -0.72 | -1.20 | -1.20 | -1.24 | -0.79 | -0.58 | -1.03 |
| **Q498** | -0.92 | -0.89 | -1.32 | -0.51 | -0.58 | NA | -0.39 | -1.09 | 0.01 | -0.41 | -0.44 | -0.96 | -0.44 | -0.38 | -1.02 | -0.74 | -0.76 | -0.27 | 1.18 | -0.51 |
| **T500** | -0.18 | -0.02 | 1.53 | 0.12 | -0.15 | -0.09 | -0.04 | -0.35 | 0.11 | -0.14 | -0.22 | -0.11 | -0.21 | -0.22 | -0.12 | 0.37 | NA | 0.02 | 0.00 | -0.22 |
| **N501** | -0.71 | -0.73 | 0.43 | -0.29 | -0.48 | -0.42 | -0.46 | -0.55 | -0.20 | 0.91 | -0.38 | -1.40 | -0.29 | -0.31 | 0.05 | -0.42 | 0.70 | -0.69 | -0.26 | 0.36 |
| **G502** | -0.01 | -0.34 | -0.11 | 0.14 | -0.08 | -0.14 | 0.07 | NA | -0.19 | -0.37 | -0.40 | -0.31 | -0.28 | 0.01 | 0.24 | -0.11 | -0.16 | 0.09 | 0.19 | -0.24 |
| **Y505** | -0.68 | -0.384 | -0.23 | -0.49 | -0.364 | -0.49 | -0.46 | -0.82 | -0.31 | -0.653 | -0.65 | -0.34 | -0.68 | 0.002 | -1.54 | -0.36 | -0.34 | -0.1 | NA | -0.24 |

**Table S22. The predicted binding stabilities of SARS-CoV-2 RBD targeting MR17 by mCSM-PPI2.**

|  | **A** | **R** | **N** | **D** | **C** | **Q** | **E** | **G** | **H** | **I** | **L** | **K** | **M** | **F** | **P** | **S** | **T** | **W** | **Y** | **V** |
| --- | --- | --- | --- | --- | --- | --- | --- | --- | --- | --- | --- | --- | --- | --- | --- | --- | --- | --- | --- | --- |
| **R403** | -0.92 | NA | -0.93 | -0.91 | -0.88 | -0.75 | -0.96 | -0.95 | -0.30 | -0.58 | -0.57 | 0.35 | -0.81 | -0.38 | -1.21 | -0.74 | -0.85 | -0.11 | -0.37 | -0.69 |
| **K417** | -0.47 | -0.24 | -0.33 | -0.58 | -0.57 | -0.51 | -0.57 | -0.70 | -0.27 | -0.35 | -0.51 | NA | -0.50 | -0.48 | -1.43 | -0.28 | -0.34 | -0.22 | -0.26 | 0.00 |
| **G446** | -0.14 | -0.35 | -0.06 | 0.17 | -0.07 | -0.01 | 0.24 | NA | -0.04 | -0.06 | -0.08 | -0.17 | -0.07 | 0.20 | -0.15 | -0.07 | -0.07 | 0.46 | 0.25 | -0.05 |
| **Y453** | -1.44 | -1.50 | -1.38 | -1.77 | -1.49 | -1.59 | -1.75 | -1.64 | -1.34 | -1.47 | -1.44 | -1.54 | -1.54 | -0.94 | -2.12 | -1.45 | -1.50 | -0.49 | NA | -1.47 |
| **L455** | -0.71 | -0.35 | -0.26 | -0.59 | -0.56 | -0.47 | -0.65 | -0.75 | -0.45 | -0.75 | NA | -0.26 | -0.82 | 0.47 | -1.03 | 0.43 | -0.22 | -0.12 | 1.22 | -0.73 |
| **F456** | -0.50 | -0.47 | -0.35 | -0.57 | -0.60 | -0.33 | -0.52 | -0.68 | 0.06 | -0.79 | -0.45 | -0.38 | -0.67 | NA | -1.44 | -0.47 | -0.45 | -0.08 | 0.38 | -0.60 |
| **I472** | -0.18 | 0.03 | -0.06 | -0.05 | -0.12 | 0.00 | 0.57 | -0.31 | -0.09 | NA | -0.10 | 0.00 | -0.22 | 0.36 | -0.24 | -0.01 | 0.02 | 0.70 | 0.36 | -0.15 |
| **E484** | -0.58 | -0.70 | -0.66 | -0.09 | -0.64 | -0.53 | NA | -0.54 | -0.12 | -0.41 | -0.51 | -0.91 | -0.54 | -0.17 | 0.53 | -0.62 | -0.66 | 0.34 | -0.02 | -0.55 |
| **G485** | -0.54 | -1.21 | -0.62 | -0.74 | -0.67 | -0.77 | -0.65 | NA | -0.65 | -0.59 | -0.85 | -1.01 | -0.79 | 0.00 | -0.88 | -0.36 | -0.59 | 0.43 | -0.01 | -0.88 |
| **F486** | -2.42 | -2.45 | -2.54 | -2.89 | -2.29 | -2.50 | -2.85 | -2.47 | -2.08 | -2.19 | -2.21 | -2.58 | -2.27 | NA | -3.34 | -2.39 | -2.44 | -0.34 | -0.98 | -2.41 |
| **N487** | -1.02 | -1.02 | NA | -0.14 | -0.92 | -0.90 | -0.95 | -1.10 | -0.54 | -0.98 | -1.02 | -0.97 | -0.98 | -0.53 | -0.77 | -0.83 | -0.88 | -0.29 | -0.44 | -0.95 |
| **C488** | -1.48 | -1.38 | -1.57 | -1.34 | NA | -1.47 | -1.32 | -1.55 | -0.90 | -1.25 | -1.11 | -1.46 | -1.34 | -0.43 | -1.70 | -1.50 | -1.54 | -0.34 | -0.26 | -1.28 |
| **Y489** | -1.34 | -0.86 | -1.13 | -1.37 | -1.19 | -1.21 | -1.32 | -1.31 | -1.17 | -1.30 | -1.36 | -1.21 | -1.32 | -0.90 | -1.69 | -1.15 | -1.15 | -0.36 | NA | -1.35 |
| **F490** | -1.48 | -1.31 | -1.27 | -1.41 | -1.24 | -1.38 | -1.28 | -1.64 | -1.51 | -1.37 | -1.40 | -1.26 | -1.40 | NA | -2.05 | -1.25 | -1.34 | 0.92 | 0.61 | -1.41 |
| **L492** | -0.73 | -0.62 | -0.65 | -1.11 | -0.91 | -0.63 | -1.09 | -0.89 | -0.20 | -0.62 | NA | -0.56 | -0.57 | 0.93 | -1.37 | -0.71 | -0.67 | 0.23 | 0.36 | -0.73 |
| **Q493** | -1.60 | -1.34 | 1.11 | -1.35 | -1.62 | NA | -1.14 | -1.70 | -0.30 | -1.36 | -1.40 | -1.44 | -1.31 | -1.11 | -1.50 | 1.25 | -1.32 | -0.75 | -0.89 | -1.42 |
| **S494** | -1.04 | -0.24 | -0.60 | 1.28 | -0.74 | -0.56 | -0.39 | -1.25 | 0.11 | -0.66 | -0.77 | -0.35 | -0.67 | -0.65 | -0.99 | NA | 1.38 | -0.37 | -0.22 | -0.62 |
| **Y495** | -1.49 | -1.48 | -1.35 | -1.75 | -1.46 | -1.58 | -1.72 | -1.71 | -1.55 | -1.47 | -1.42 | -1.46 | -1.47 | -1.05 | -2.09 | -1.41 | -1.40 | -0.32 | NA | -1.45 |
| **Q498** | -0.44 | -0.10 | 0.21 | -0.25 | -0.42 | NA | -0.15 | -0.48 | 0.09 | -0.27 | -0.26 | -0.23 | -0.27 | 0.04 | -0.69 | -0.31 | -0.31 | -0.06 | 1.04 | -0.31 |
| **Y505** | -0.53 | -0.42 | -0.19 | -0.60 | -0.35 | -0.42 | -0.59 | -0.62 | -0.23 | -0.50 | -0.60 | -0.37 | -0.54 | -0.11 | -1.35 | -0.33 | -0.34 | -0.01 | NA | -0.53 |

**Table S23. The predicted binding stabilities of SARS-CoV-2 RBD targeting P2B-2F6 by mCSM-PPI2.**

|  | **A** | **R** | **N** | **D** | **C** | **Q** | **E** | **G** | **H** | **I** | **L** | **K** | **M** | **F** | **P** | **S** | **T** | **W** | **Y** | **V** |
| --- | --- | --- | --- | --- | --- | --- | --- | --- | --- | --- | --- | --- | --- | --- | --- | --- | --- | --- | --- | --- |
| **R346** | -0.27 | NA | -0.18 | -0.34 | -0.29 | -0.11 | -0.36 | -0.28 | -0.05 | -0.22 | -0.25 | 0.19 | -0.34 | -0.02 | -0.82 | -0.21 | -0.20 | 0.06 | 0.02 | -0.33 |
| **Y351** | -0.36 | -0.29 | -0.29 | -0.45 | -0.33 | -0.41 | -0.42 | -0.48 | -0.47 | -0.38 | -0.40 | -0.34 | -0.41 | -0.07 | -0.98 | -0.35 | -0.32 | 0.28 | NA | -0.38 |
| **V445** | -0.03 | -0.09 | -0.06 | -0.13 | -0.32 | -0.12 | -0.11 | -0.26 | 0.07 | 0.01 | 0.01 | -0.01 | -0.05 | 0.35 | 0.02 | 0.18 | 0.01 | 0.82 | 0.46 | NA |
| **G447** | -0.86 | -1.59 | -0.93 | -0.95 | -1.20 | -1.40 | -1.10 | NA | -1.20 | -1.69 | -1.67 | -1.46 | -1.46 | -1.27 | -1.49 | -0.84 | -1.17 | -1.32 | -1.11 | -1.36 |
| **N448** | -1.38 | -1.30 | NA | -0.47 | -1.35 | 0.63 | -1.27 | -1.24 | -0.18 | -0.99 | -1.09 | -1.42 | -1.24 | -1.00 | -0.93 | -1.29 | -1.18 | -0.97 | -0.51 | -1.23 |
| **Y449** | -1.89 | -1.87 | -1.71 | -2.26 | -1.70 | -2.02 | -2.15 | -2.26 | -1.49 | -1.86 | -1.76 | -1.96 | -1.88 | -0.35 | -3.04 | -1.95 | -1.87 | -0.63 | NA | -1.83 |
| **N450** | -0.67 | -0.70 | NA | -0.03 | -0.60 | -0.63 | -0.57 | -0.57 | -0.26 | -0.60 | -0.61 | -0.81 | -0.75 | -0.35 | -0.42 | -0.71 | -0.61 | -0.13 | -0.71 | -0.58 |
| **L452** | -0.91 | -0.51 | -0.81 | -1.17 | -0.97 | -0.82 | -1.38 | -0.96 | -0.40 | -0.80 | NA | 0.01 | -0.89 | 0.84 | -0.78 | -0.71 | -0.65 | 1.10 | 0.69 | -0.91 |
| **I472** | -0.40 | -0.32 | -0.25 | -0.18 | -0.38 | -0.11 | 0.22 | -0.40 | -0.42 | NA | -0.45 | -0.24 | -0.46 | 0.65 | -0.42 | -0.28 | -0.29 | 1.01 | 0.33 | -0.30 |
| **V483** | -0.11 | -0.52 | -0.43 | -0.31 | -0.35 | -0.29 | -0.21 | -0.19 | 0.03 | 0.02 | 0.02 | -0.15 | -0.22 | 0.42 | -0.14 | -0.34 | -0.31 | 0.43 | 0.31 | NA |
| **E484** | -0.48 | -0.21 | -0.45 | -0.09 | -0.53 | -0.27 | NA | -0.42 | 0.05 | -0.19 | -0.28 | -0.38 | -0.26 | 0.08 | 0.17 | -0.40 | -0.40 | 0.52 | 0.11 | -0.39 |
| **G485** | -0.14 | -0.46 | -0.09 | 0.05 | -0.13 | -0.06 | 0.09 | NA | -0.10 | -0.01 | -0.10 | -0.29 | -0.05 | 0.12 | -0.22 | -0.06 | -0.12 | 0.42 | 0.15 | -0.04 |
| **F486** | -0.27 | -0.82 | -0.73 | -1.13 | -0.66 | -0.68 | -1.17 | -0.26 | -0.13 | -0.48 | -0.52 | -0.68 | -0.55 | NA | -0.29 | -0.59 | -0.60 | 0.21 | 0.04 | -0.38 |
| **F490** | -1.35 | -1.36 | -1.27 | -1.89 | -1.35 | -1.50 | -1.65 | -1.73 | -1.22 | -1.19 | -1.23 | -1.43 | -1.34 | NA | -2.23 | -1.32 | -1.33 | 0.88 | 0.50 | -1.28 |
| **S494** | -0.18 | 0.18 | 0.04 | 0.96 | -0.16 | -0.05 | -0.08 | -0.25 | 0.46 | -0.04 | -0.05 | 0.03 | -0.05 | -0.02 | 0.13 | NA | 0.62 | 0.05 | 0.16 | -0.04 |

**Table S24. The predicted binding stabilities of SARS-CoV-2 RBD targeting ACE2 by mCSM-PPI2.**

|  | **A** | **R** | **N** | **D** | **C** | **Q** | **E** | **G** | **H** | **I** | **L** | **K** | **M** | **F** | **P** | **S** | **T** | **W** | **Y** | **V** |
| --- | --- | --- | --- | --- | --- | --- | --- | --- | --- | --- | --- | --- | --- | --- | --- | --- | --- | --- | --- | --- |
| **K741** | -0.60 | -0.47 | -0.47 | -0.67 | -0.56 | -0.56 | -0.65 | -0.72 | 0.08 | -0.26 | -0.42 | NA | -0.64 | -0.14 | -1.08 | -0.50 | -0.44 | -0.11 | -0.04 | 0.21 |
| **G446** | -0.22 | -0.51 | -0.18 | 0.02 | -0.16 | -0.17 | 0.10 | NA | -0.32 | -0.21 | -0.22 | -0.30 | -0.19 | -0.02 | -0.54 | -0.27 | -0.03 | 0.15 | 0.02 | -0.14 |
| **Y449** | -0.66 | -0.48 | -0.29 | -0.74 | -0.41 | -0.46 | -0.67 | -0.80 | -0.62 | -0.69 | -0.69 | -0.41 | -0.74 | -0.29 | -1.96 | -0.42 | -0.42 | -0.31 | NA | -0.65 |
| **Y453** | -0.85 | -0.77 | -0.73 | -0.99 | -0.89 | -0.96 | -0.96 | -1.02 | -0.76 | -0.83 | -0.82 | -0.85 | -0.91 | -0.08 | -1.62 | -0.88 | -0.90 | 0.04 | NA | -0.82 |
| **L455** | -1.55 | -1.29 | -1.22 | -1.94 | -1.38 | -1.31 | -1.97 | -1.60 | -0.74 | -0.96 | NA | -1.25 | -1.33 | 0.69 | -1.33 | 0.30 | -1.05 | -0.02 | 1.16 | -1.28 |
| **F456** | -1.63 | -1.68 | -1.31 | -1.85 | -1.59 | -1.23 | -1.82 | -1.69 | 0.28 | -1.46 | -1.19 | -1.56 | -1.40 | NA | -1.39 | -1.41 | -0.42 | 0.61 | -1.45 | NA |
| **A475** | NA | 0.36 | 0.56 | 1.03 | 0.30 | 0.43 | 0.70 | -0.36 | 0.35 | 0.27 | 0.18 | 0.40 | -0.09 | 1.14 | -0.35 | 0.17 | 0.35 | 1.46 | 1.08 | 0.00 |
| **F486** | -0.78 | -0.78 | -0.68 | -1.05 | -0.81 | -0.72 | -1.05 | -0.80 | -1.09 | -0.94 | -0.94 | -0.83 | -0.97 | NA | -1.01 | -0.60 | -0.66 | -0.27 | -0.60 | -0.85 |
| **N487** | -1.00 | -0.93 | NA | -0.30 | -1.10 | -1.00 | -0.76 | -1.01 | 0.05 | -0.52 | -0.61 | -1.12 | -0.59 | -0.27 | -0.30 | -0.87 | -1.01 | -0.01 | -0.23 | -0.57 |
| **Y489** | -1.95 | -1.91 | -1.87 | -2.37 | -1.94 | -2.00 | -2.33 | -1.94 | -1.76 | -1.70 | -1.79 | -1.86 | -1.90 | -1.05 | -2.00 | -1.90 | -1.91 | -0.41 | NA | -1.90 |
| **Q493** | -0.69 | -0.58 | 0.62 | -0.35 | -0.58 | NA | -0.08 | -0.60 | 0.25 | -0.37 | -0.38 | -0.81 | -0.37 | -0.18 | -0.24 | 0.66 | -0.46 | -0.12 | 0.03 | -0.40 |
| **G496** | -0.50 | -1.37 | -1.17 | 1.03 | -1.16 | -1.24 | -0.87 | NA | -0.43 | -1.14 | -1.17 | -1.42 | -1.07 | -0.40 | -1.28 | -0.98 | -1.14 | -0.37 | -0.27 | -0.91 |
| **Q498** | -1.44 | -1.36 | 1.16 | -0.96 | -1.11 | NA | -1.00 | -1.55 | -0.53 | -0.97 | -1.05 | -1.48 | -1.01 | -0.31 | -1.94 | -1.22 | -1.22 | -0.56 | 2.00 | -0.95 |
| **T500** | -0.58 | -0.59 | 0.75 | -0.53 | -0.66 | -0.58 | -0.66 | -0.73 | -0.24 | -0.52 | -0.60 | -0.49 | -0.56 | -0.41 | -0.51 | 0.24 | NA | -0.34 | -0.26 | -0.55 |
| **N501** | -1.17 | -1.02 | NA | -0.64 | -0.87 | -0.83 | -0.85 | -0.97 | -0.26 | 1.25 | -0.92 | -1.27 | -0.80 | -0.69 | -1.04 | -0.88 | 0.88 | -0.84 | -0.54 | 0.27 |
| **G502** | -0.77 | -1.31 | -0.92 | -0.97 | -1.16 | -1.04 | -1.01 | NA | -0.91 | -1.29 | -1.30 | -1.23 | -1.19 | -0.99 | 0.94 | -0.61 | -1.14 | -0.91 | -0.90 | -1.10 |
| **Y505** | -1.36 | -1.30 | -1.04 | -1.57 | -1.15 | -1.36 | -1.44 | -1.54 | -1.03 | -1.23 | -1.27 | -1.32 | -1.36 | -0.27 | -2.31 | -1.27 | -1.18 | -0.47 | NA | -1.35 |
